# Effect of Thiophene Insertion on X-Shaped Anthracene-Based Hole-Transporting Materials in Perovskite Solar Cells

**DOI:** 10.3390/polym14081580

**Published:** 2022-04-13

**Authors:** Dharuman Chandrasekaran, Wei-Hao Chiu, Kun-Mu Lee, Jian-Ming Liao, Hsien-Hsin Chou, Yung-Sheng Yen

**Affiliations:** 1 Department of Chemistry, Chung Yuan Christian University, Zhongli, Taoyuan 320, Taiwan; chandrugood91@gmail.com; 2 Center for Reliability Sciences and Technologies, Chang Gung University, Guishan, Taoyuan 333, Taiwan; weihao.chiu@gmail.com; 3 Department of Chemical and Materials Engineering, Chang Gung University, Guishan, Taoyuan 333, Taiwan; 4 Center for Green Technology, Chang Gung University, Guishan, Taoyuan 333, Taiwan; 5 Division of Neonatology, Department of Pediatrics, Chang Gung Memorial Hospital, Linkou, Taoyuan 33305, Taiwan; 6 Department of Applied Chemistry, Providence University, Taichung 433, Taiwan; jl387555@gmail.com

**Keywords:** anthracene, tetra-substituted, perovskite solar cells, hole-transporting materials

## Abstract

In this work, two novel tetra-substituted **X**-shaped molecules **X1** and **X2** that were constructed with anthracene as the central core and arylamine as the donor groups have been synthesized. The HTMs **X1** and **X2** were synthesized in two steps from industrially accessible and moderately reasonable beginning reagents. These new HTMs are described in terms of utilization of light absorption, energy level, thermal properties, hole mobility (µ_h_), and film-forming property. The photovoltaic performances of these HTMs were effectively assessed in perovskite solar cells (PSCs). The devices based on these HTMs accomplished an overall efficiency of 16.10% for **X1** and 10.25% for **X2** under standard conditions (AM 1.5 G and 100 mW cm^−2^). This precise investigation provides another perspective on the use of HTMs in PSCs with various device configurations.

## 1. Introduction

Sun-oriented energy for an economical future has inspired the development of new advancements, including photovoltaic green advancements such as sunlight-based cells and batteries that are harmless to the ecosystem. Perovskites were presented as dynamic materials for photovoltaic applications by Miyasaka and colleagues in 2009 [1]. The photovoltaic performance of perovskite solar cells (PSCs) has achieved remarkable improvement, producing a significant increase from 3.9% in 2009 to 22.1% [2] in recent years, and a record of 25.2% has been reported. In perovskite-solar-cell (PSC) devices, light absorption by the perovskite material prompts the generation of excitons (hole and electron pair), which should be productively isolated for a high quantum yield [3,4]. In general, the perovskite material is sandwiched by two specific layers of n-type/p-type semiconductors in PSC devices. A typical PSC is comprised of an electron-transport layer (ETL) [5], a semiconducting perovskite material, a hole-transport layer (HTL) [6], and a metal-coated counter electrode. Hole-transporting materials (HTMs) play a significant part in exploiting the photo-generated hole-extraction and collection capacity, suppressing electronic recombination measures, and reducing the perovskite debasement by interfacial change [7,8,9,10,11]. In PSCs, hole-transporting materials (HTMs) play an important role in achieving high power-conversion efficiency. Currently, two unique primary kinds of HTMs have been applied along with organic and inorganic p-type semiconductors [12,13,14,15,16]. A wide range of conjugated, small organic molecules and polymers, as well as inorganic compounds, is under dynamic investigation for next-generation HTMs in PSCs [17,18,19,20,21,22,23,24,25,26]. Compared to other HTMs, small molecule-based semiconductors are particularly useful considering they facilitate easy synthesis and purification, as well as good film-forming properties during device fabrication [6]. Amongst small molecular HTMs, compounds with planar and inflexible skeletons are promising due to effective intermolecular π stacking, which facilitates hole transport [27,28,29]. 

The most widely used tactic to develop a large number of D-π-D types of HTMs is to change the π-linker, due to its relatively simple molecular structure. The preferred *2*,*2′*,*7*,*7′*-tetrakis-(*N*,*N*-di-*p*-methoxy-phenyl-amine)*9*,*9′*-spirobifluorene (Spiro-OMeTAD) is the most recognized and regularly utilized small-particle HTM for PSCs, and it exhibits great power-conversion efficiency (PCE). Unfortunately, the synthesis of spiro-OMeTAD materials includes convoluted response conditions and costly purification procedures [30]. In any case, the advancement of a low-cost and productive HTM to replace the costly spiro-OMeTAD is needed in the commercial utilization of PSCs [31,32]. Among them, the small organic molecules with multi-branched molecular skeletons that easily form amorphous morphology and supply more pathways for hole hopping [25,33,34], for example, pyrene [21], dibenzo[*g*,*p*]chrysene [35], *9*,*9’*-bifluorenylidene [36], thieno [*3*,*2*-*b*]thiophenes [37] benzodithiophene [38], acridine [39], and their derivatives, etc., have attracted significant attention due to the benefits of their optoelectronic properties. So far, a couple of them can accomplish practically identical photovoltaic performance to spiro-OMeTAD with PCEs around 21% [2,40,41,42,43,44,45]. 

Anthracene possesses an electron-donating ability and better planarity for further development of the primary assisted charge transport [46,47]. Past reports have focused on the creation of *9*,*10*-substituted anthracene derivatives and their use as blue-light-transmitting materials for OLED devices on account of their tight bandgap and strong blue fluorescence [48,49,50], as well as organic thin-film semiconductors [51,52], dye-sensitized solar cells (DSSCs) [53,54], and bulk heterojunction organic solar cells [55] due to its greater carrier mobility, high stability, and ease of modification. Recently, Prof. Dai et al. reported two HTMs (A101, and A102) by incorporating diarylamine or triarylamine into a *9*,*10*-linked anthracene-based small molecule, and PSCs based on A102 yielded a PCE of 17.56%, which is higher than that of spiro-OMeTAD (17.27%) [56]. In another example, Prof. Sonar et al. designed and synthesized diarylamine or triarylamine to *2*,*6*-anthracene-based HTMs, i.e., inverted-type PSCs with p–i–n structures, and the PSCs attained a PCE of 7.54% for TPA-ANR-TPA, and 6.05% for DPA-ANR-DPA [57]. *2*,*6*-substituted anthracene-based small-molecule HTMs [57,58] or polymers [59] also have better hole mobility and hole-extraction capacity, leading to the production of high PCEs. Very recently, we reported novel HTMs based on dihydrodinaphthopentacene (DHDNP) and found that polyaromatic hydrocarbon DHDNP-based HTMs showed high mobility and a comparable PCE with spiro-OMeTAD [60]. In our continued research on HTMs with polyaromatic hydrocarbons, we decided to explore the *2*,*6*,*9*,*10*-substituent small anthracene molecules which joined bis(*4*-methoxyphenyl)amine) as the donor group based on the following reasons: (1) the arylamine moiety may benefit hole-hopping [19,61]; (2) the planar structure of anthracene might assist with intermolecular stacking, prompting expanded intermolecular electronic coupling; (3) the anthracene moiety is electron-rich and may function as the second electron contributor. Heterocyclic thiophene has been widely used in optoelectronic materials due to the electron-rich nature of sulfur and the coplanar π-conjugated framework, which can enhance the hole mobility through an intermolecular overlap of π-π clouds in the solid state. Not only the π-π interaction, but the S···S interaction is also beneficial to charge transport due to the electronic softness of the sulfur atoms and, thus, an increase in effective overlapping. These impacts have been exhibited by many thiophene-containing aromatic compounds [21,35,37,62]. Therefore, we also inserted the thienyl group into the molecular configuration to explore the impact on the devices.

In this study, we present two novel HTMs (**X1**, **X2**) comprising an anthracene central core with four electroactive arylamine moieties attached to its frame. Compared with the synthesis of the spiro-OMeTAD system, the synthesis of *2*,*6*,*9*,*10*-tetrabromoanthracene is a moderate-yield, one-step procedure using inexpensive commercial materials. The absorption properties, energy levels, thermal analysis, hole-transport properties, and film-forming properties of the two new anthracene-based compounds were investigated. The two new HTMs show good light-absorption ability and appropriate hole mobility. The PSC fabrication using **X1** and **X2** as HTMs will also be discussed.

## 2. Materials and Methods

### 2.1. General Method

All other solvents and chemicals were purchased from Aldrich (St. Louis, MO, USA), and the purity of all commercial materials and solvents was more than 98%. Unless otherwise specified, all reactions and manipulations were carried out under a nitrogen atmosphere. Solvents THF, toluene, and DMF were dried by standard procedures. All chromatographic separations were carried out on silica gel (60 M, 230–400 mesh). Compound **1** was synthesized according to the literature method [63]. Bis(*4*-methoxyphenyl)amine (**2**) [64], and *N*,*N*-bis(*4*-methoxyphenyl)-*5*-(tributylstannyl)thiophen-*2*-amine (**3**) [65], were prepared according to the procedures described in the literature method.

### 2.2. Electrochemical Characterization

Cyclic voltammetry was carried out with an electrochemical workstation (CHI 611E, CH Instruments, Inc., Bee Cave, TX, USA) using a conventional three-electrode configuration. A three-electrode system consisting of a glassy-carbon working electrode, an Ag/AgNO_3_ reference electrode, and a Pt-wire counter electrode was used. The redox potential of the materials was measured in dichloromethane solution with 0.1 M (n-C_4_H_9_)_4_NPF_6_ as the supporting salt at a scan rate of 100 mVs^−1^. Potentials were calibrated with respect to the Fc^+^/Fc couple.

### 2.3. Optical Characterization

Absorption and emission spectra were recorded on a Jasco V-730 UV–Vis spectrophotometer (Japan Spectroscopic Company, Tokyo, Japan) and Jasco FB-8300 spectrofluorometer (Japan Spectroscopic Company, Tokyo, Japan). All samples were measured at room temperature with dichloromethane as the solvent. The concentration of 1 × 10^−5^ mol mL^−1^ was used for the UV–Vis and PL solutions, respectively. 

### 2.4. Thermal Characterization 

The decomposition temperature was carried out on a Thermal Gravimetric Analyzer (TGA, Q50, TA Instruments, Chicago, IL, USA). Differential scanning calorimetry (DSC) was performed on NETZSCH DSC 214 Polyma DSC21400A-0324-L at a scan rate of 20 °C/20.0 (K/min)/371 °C under nitrogen atmosphere in DSC/TG aluminum pan. Melting points were determined by using Buchi melting point B-540 (BUCHI Corp., Lukens Drive, New Castle, DE, USA).

### 2.5. Device Fabrication and Characterization 

The PSCs were fabricated with the structure of ITO/SnO_2_/perovskite/HTM/Ag. The ITO substrate (7 Ω/square, Ruilong Optoelectronics, Miaoli, Taiwan) was washed by subsequent sonication steps in acetone, ethanol, isopropanol, and DI water, respectively. To remove organic residues from the surface, the ITO substrate was treated with UV-ozone cleaner for 15 min. The electron-transport layer, SnO_2_, was deposited by blade coating on the ITO glass with commercial SnO_2_ solution (dilute to 7%) (Alfa Aesar, Harverhill, MA, USA) at 60 °C (coating speed: 10 mm/s, gap: 200 μm) and baked on a hot plate at 155 °C for 40 min. Then, the perovskite (Cs_0.05_MA_0.2_FA_0.75_Pb(Br_0.05_I_0.95_)_3_) layer was produced using a single-step method. The mixture of 576.3 mg of PbI_2_ (99.9985%, Alfa Aesar (Harverhill, MA, USA), 161.2 mg of formamidinium iodide (FAI, 99.99%, FMPV^®^, FrontMaterials Co. Ltd., Taipei, Taiwan), 14 mg of methylammonium bromide (MABr, ECHO Chemical, Miaoli, Taiwan), 16.2 mg of Cesium iodide (CsI, Aldrich, Burlington, VT, USA) and 19.8 mg of methylammonium iodide (CH_3_NH_3_I, MAI, >98%, STAREK^®^, Starek Scientific Co. Ltd., Taipei, Taiwan) dissolved in 0.8 mL dimethylformamide (DMF, Tedia, OH, USA) and 0.2 mL Dimethyl sulfoxide (DMSO, 99%, Echo Chemical Co. Ltd., Miaoli, Taiwan) was stirred at 60 °C for 8 h. This solution was then spin coated on the compact SnO_2_ film at 1000 rpm for 10 s and 5000 rpm for 20 s and then baked on a hot plate for 10 min. Subsequently, the hole-transporting layer was spin coated at 2000 rpm for 30 s by using different HTM solutions. The HTM solutions were composed of HTM (Spiro-OMeTAD (99.93%, Rruilong, Miaoli, Taiwan): 80 mg, **X1**: 50 mg, **X2**: 50 mg), 17.5 μL lithium bis(trifluoromethanesulfonyl)imide (LiTFSI, 99.9%, Sigma-Aldrich, Burlington, VT, USA) and 28.5 μL *4*-tert-butyl pyridine (*4*-TBP, Aldrich, Burlington, VT, USA), 1 mL chlorobenzene (CB, 99%, Tedia, OH, USA) under N_2_ conditions. To complete the PSC production, a 100 nm silver electrode was thermally evaporated as the top electrode with a 0.09 cm^2^ mask to define the active area of the device.

The surface morphology of the PSCs was measured through scanning electron microscopy (SEM) with a field-emission scanning electron microscope (Hitachi SU8010, Tokyo, Japan; acceleration voltage, 5 kV; working distance, 8.4 mm). Surface morphologies of samples were recorded by a tapping-mode atomic-force microscope (AFM) (Nano-Scope NS3A System, Digital Instruments, Bresso, Italy). The photoluminescence (PL) spectra were recorded with a 532 nm diode laser (LDH-D-TA-530, PicoQuant, Berlin, Germany). 

The measurement procedures for the *J*–*V* curves and incident-photon-to-current-efficiency (IPCE) spectra were as follows. The cell parameters were obtained under incident light with an AM 1.5 global filter generated by a Xe arc lamp. The light intensity was further calibrated using an NREL certified silicon diode with an integrated KG5 with <5% spectral mismatch factors. The power-conversion efficiencies of the forward scan (FS) and reverse scan (RS) were recorded with biased voltage from J_SC_ to V_OC_ and from V_OC_ to J_SC_, respectively. The monochromatic quantum efficiency was recorded using commercial IPCE set-up (QE-R, Enlitech, Kaohsiung, Taiwan) under short-circuit conditions with DC mode and without any light bias. 

### 2.6. Mobility Measurements

To assess the potential HTMs **X1**, **X2**, and spiro-OMeTAD as hole-transport materials, hole-only devices were fabricated by using the space-charge-limited-current (SCLC) method with the device structure ITO/PEDOT: PSS/HTM/Ag. Hole mobilities were calculated using the Mott–Gurney law, by fitting Equation (1) to experimental data in the voltage range where the obtained slope in the double log plot is equal to 2.

(1) $$J=\frac{9}{8}\varepsilon_{r}\varepsilon_{0}\mu h\frac{v^{2}}{d^{3}}$$

In Equation (1), J is the current density, ε_0_ is the permittivity of free space (8.85 × 10^−12^ F m^−1^), ε_r_ is the relative permittivity of the material (approaching 3 for organic semiconductors), μ_h_ is the hole mobility, V is the applied voltage and d is the thickness of the active layer. The devices for SCLC measurement were fabricated by spin coating PEDOT: PSS (Clevios P, VP Al4083) onto pre-cleaned, patterned indium-tin-oxide (ITO) substrates (7 Ω/square, Ruilong Optoelectronics, Miaoli, Taiwan). The HTM solution that was the same as the solution used to fabricate the solar cell was spin coated on top of PEDOT:PSS. A silver electrode was deposited by thermal evaporation. The current-density–voltage curves of the devices were recorded with a Keithley 2400 source.

### 2.7. Synthesis

#### 2.7.1. Synthesis of *N^2^,N^2^,N^6^,N^6^,N^9^,N^9^,N^10^,N^10^*-Octakis(4-methoxyphenyl)anthracene-2,6,9,10-tetraamine (**X1**)

Compound **1** (1.0 g, 2.02 mmol), compound **2** (1.85 g, 8.10 mmol), Pd(dba)_2_ (0.093 g, 0.162 mmol), sodium tert-butoxide (1.16 g, 12.15 mmol), (t-Bu)_3_P (0.49 M, 0.66 mL, 0.324 mmol) were dissolved in dry toluene (30 mL) under N_2_. The reaction mixture was heated to 90 °C for 4 days. After it cooled to room temperature, the mixture was extracted with dichloromethane/water. After drying over anhydrous MgSO_4_ the organic solvents were evaporated, and the crude product was purified by column chromatography on silica gel using dichloromethane/hexane (1.5:1) as the eluent. Subsequent precipitation from hexane gave the product **X1** as a dark orange solid (1.1 g, 50% yield). ^1^H NMR (400 MHz, CDCl_3_): δ 7.78 (d, J = 9.6 Hz, 2H), 7.34 (s, 2H), 6.96 (dd, J = 9.6 Hz, 2.4 Hz, 2H), 6.90 (d, J = 8.8 Hz, 8H), 6.78 (d, J = 9.2 Hz, 8H), 6.65 (m, 16H), 3.76 (d, J = 6.4 Hz, 24H). ^13^C NMR (100 MHz, CDCl_3_): δ 155.88, 153.55, 145.37, 142.04, 140.32, 126.81, 125.29, 123.12, 121.06, 114.63, 114.41, 55.50, 55.43. Anal. cald. for (C_70_H_62_N_4_O_8_): C, 77.33%; H, 5.75%; N, 5.15% Found C, 77.55%; H, 5.82%; N, 5.26%. mp: 328 °C.

#### 2.7.2. 5-(6,9,10-tris(5-(bis(4-Methoxyphenyl)amino)thiophen-2-yl)anthracen-2-yl)-*N*,*N*-bis(4-methoxyphenyl)thiophen-2-amine (**X2**)

Compound **1** (1.0 g, 2.02 mmol), compound **3** (6.079 g, 10.12 mmol), PdCl_2_(PPh_3_)_2_ (0.142 g, 0.202 mmol), were dissolved in dry and deoxygenated DMF (30 mL) under N_2_. The reaction mixture was heated to 90 °C for 4 days. After it cooled to room temperature, aqueous KF was added, and the mixture was extracted with dichloromethane/water. After drying over anhydrous MgSO_4_ the organic solvents were evaporated, and the crude product was purified by column chromatography on silica gel using dichloromethane/Hexane (1.5:1) as the eluent. Subsequent precipitation from hexane gave the product **X2** as a dark red solid (0.925 g, 46.04% yield). ^1^H NMR (400 MHz, CDCl_3_): δ 8.00 (d, J = 7.2 Hz, 4H), 7.58 (dd, J = 9.2 Hz, 1.6 Hz, 2H), 7.15 (m, 16H), 7.12 (d, J = 4 Hz, 2H), 6.87 (d, J = 4 Hz, 2H), 6.81 (m, 16H), 6.66 (d, J = 3.6 Hz, 2H), 6.48 (d, J = 4 Hz, 2H), 3.77 (d, J = 3.6 Hz, 24H). ^13^C NMR (100 MHz, CDCl_3_): δ 155.95, 155.74, 155.68, 154.77, 153.69, 141.74, 141.34, 135.29, 131.75, 131.27, 131.05, 129.85, 129.83, 128.41, 127.27, 124.72, 124.69, 124.59, 124.44, 124.39, 123.91, 122.61, 121.17, 117.76, 117.06, 114.61, 114.60, 55.53, 55.50. Anal. cald. for (C_86_H_70_N_4_O_8_S_4_): C, 72.96%; H, 4.98%; N, 3.96% Found C, 73.18%; H, 5.16%; N, 4.14%. mp: 178 °C.

## 3. Results and Discussion

### 3.1. Synthesis Materials

**Scheme 1:**
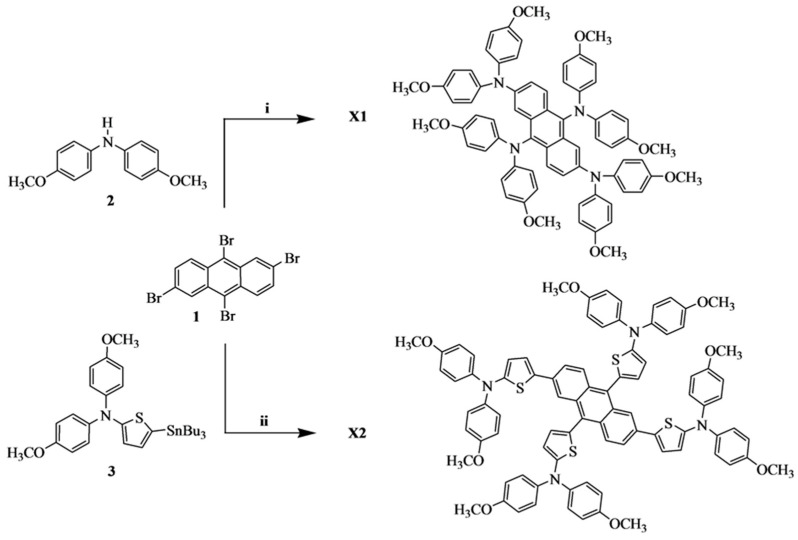
Synthesis of **X1**, **X2** (i) Pd(dba)_2_, sodium tert-butoxide, tri-tert-butylphosphine, toluene. (ii) PdCl_2_(PPh_3_)_2_, DMF.

The molecular structures and the synthetic routes of these two HTMs, **X1** and **X2,** are depicted in Scheme 1. All reactions were carried out under a nitrogen atmosphere. The starting material 2,6,9,10-tetrabromoanthracene (**1**) was synthesized by the literature procedure [19]. The addition of the bis(4-methoxyphenyl)amino component can guarantee the dissolvability of the materials in organic solvents, which is essential for their homogeneous film morphology and photovoltaic performances [66]. **X1** was obtained from the reaction of compound **1** with bis(4-methoxyphenyl)amine by palladium-catalyzed Buchwald–Hartwig cross-coupling reaction. In the meantime, **X2** was prepared from the Stille cross-coupling reaction of compound **1** with stannyl compound **3**. These materials were completely characterized with ^1^H NMR spectroscopy and ^13^C NMR spectroscopy.

### 3.2. Optical Properties

**Figure 1:**
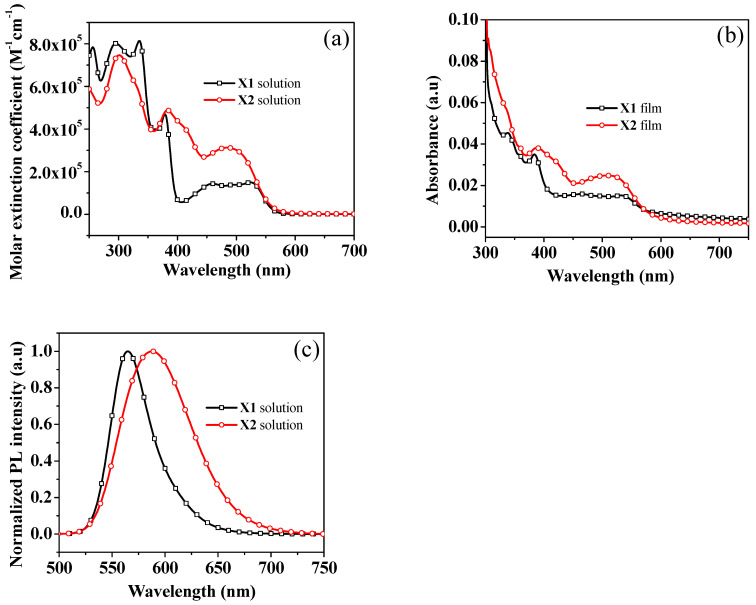
(**a**) UV–Vis absorption spectra of **X1**, **X2** in DCM solution; (**b**) Absorption spectra of **X1**, **X2** recorded in film state; (**c**) Emission spectra of **X1**, **X2** in DCM solution.

The photophysical properties of **X1** and **X2** were investigated. Figure 1 shows the UV–Vis absorption and fluorescence (FL) spectra of these compounds in DCM (1 × 10^−5^ M) solution and as solid films, and the resulting data are summarized in Table 1. The absorption spectra of **X1** and **X2** display absorption bands around 300–580 nm, as shown in Figure 1a. The shorter wavelength peaks at 300–400 nm are attributed to the localized π-π∗ electronic transition. The lower-energy absorption peaks of **X1** and **X2** were 523 nm (ε = 1.49 × 10^5^ M^−1^ cm^−1^) and 486 nm (ε = 3.12 × 10^5^ M^−1^ cm^−1^), respectively, which are ascribed to the intramolecular charge transfer (ICT) with a more delocalized π-π* transition character. The λ_max_ of the ICT band in **X1** being larger than that of **X2** indicates the stronger intramolecular charge transfer of **X1** than that of **X2**. The absorption spectra of **X1** and **X2** in the thin-film state (Figure 1b) show a bathochromic shift of 10–39 nm compared with the solutions, which is due to the intermolecular interaction in the films. The photoluminescence spectra of **X1** and **X2** are shown in Figure 1c. The maximum emission peak of **X1** and **X2** was observed at 564 and 589 nm with Stokes shift of 34 and 103 nm, respectively. The Stokes shifts between the absorption and the emission bands were likewise supported for charge-transfer characteristics in both molecules. The optical band-gap energies acquired from the intersection of absorption and emission spectra of **X1** and **X2** were 2.28 and 2.31 eV, respectively. The absorption and emission spectra of compounds **X1** and **X2** in variant solvents are displayed in Figure S1. No obvious solvent effect was observed on the absorption of **X1** or **X2** in various solvents. The emission bands of **X1** and **X2** were slightly sensitive to solvent polarity. The emission bands showed a slightly bathochromic shift for **X1** and **X2** in high-polarity solvents, which indicates the existence of twisted intramolecular-charge-transfer (ICT) excited states [67]. Both the synthesized HTMs displayed large absorption and emission spectra ranges with large Stokes-shift values as compared with those of the reference compound spiro-OMeTAD. 

### 3.3. Electrochemical Properties

**Figure 2:**
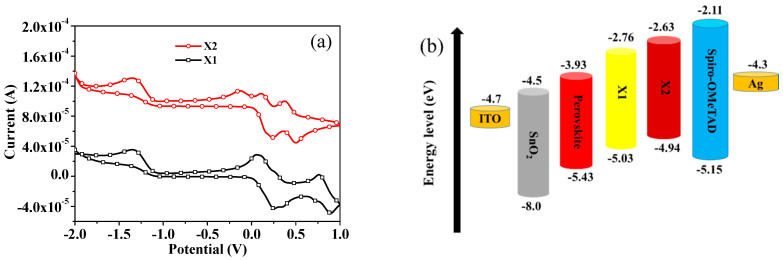
(**a**) Cyclic-voltammogram curves of compounds **X1**, **X2**, spiro-OMeTAD in DCM solution; (**b**) The energy-level diagram of **X1**, **X2**, spiro-OMeTAD, and CH_3_NH_3_PbI_3_.

The electrochemical properties of the new HTMs were studied utilizing cyclic voltammetry (CV), which is displayed in Figure 2a. The HOMO and LUMO energy levels of these mixtures were estimated by cyclic voltammetry in dichloromethane (1 × 10^−3^ M) at 25 °C using tetrabutylammonium hexafluorophosphate as the electrolyte. All redox potentials were referenced to ferrocene, which was utilized as an internal standard for calibrating the potential and calculating the HOMO levels. The redox peaks of **X1** and **X2** were both quasi-reversible, indicating that these two HTMs have good electrochemical stability. The HOMO of **X1** and **X2** was determined as −5.03 and −4.94 eV, respectively, which is higher than that of the perovskite valence band (ca. −5.43 eV) [60]. This demonstrates that it can provide sufficient driving force to the injection of holes from the perovskite into HTML. The open-circuit voltage in the PSCs was determined from the difference between the Fermi level of TiO_2_ and the HOMO of the HTMs [68]. The HOMO of **X2** being higher than that of **X1** is due to inserting the electron-rich thiophene moiety between the diarylamine and anthracene, and the lower HOMO of **X1** might assist with carrying out a higher open-circuit voltage in the **X1**-based PSC device. In correlation, the HOMO energy level of spiro-OMeTAD was −5.15 eV [60]. The schematic energy levels of **X1**, **X2**, and spiro-OMeTAD as an HTM are shown in Figure 2b.

### 3.4. Thermal Properties

The fundamental thermal properties of the compounds were obtained from thermogravimetric-analysis (TGA) and differential-scanning-calorimetry (DSC) measurements, and the collected data are shown in Table 1 and Figure S3. As shown in Figure S3a, the decomposition temperature (T_d_) of **X1**, **X2**, and spiro-OMeTAD [60] were recorded as 424 °C, 422 °C, 422 °C (5% weight loss), respectively, indicating that these two new HTMs have similar thermal stability to spiro-OMeTAD. Thermal transitions were studied by DSC and compared with those for spiro-OMeTAD (Figure S3b). **X2** exhibited different behavior compared to **X1** and spiro-OMeTAD in DSC. The glass-transition temperatures (T_g_) were observed at 135 °C, 95 °C, and 129 °C for **X1**, **X2**, and spiro-OMeTAD [60], respectively. The higher T_g_ of **X1** indicates a more stable amorphous state, which is comparable with spiro-OMeTAD and beneficial to the device stability.

### 3.5. Theoretical Approach

**Figure 3:**
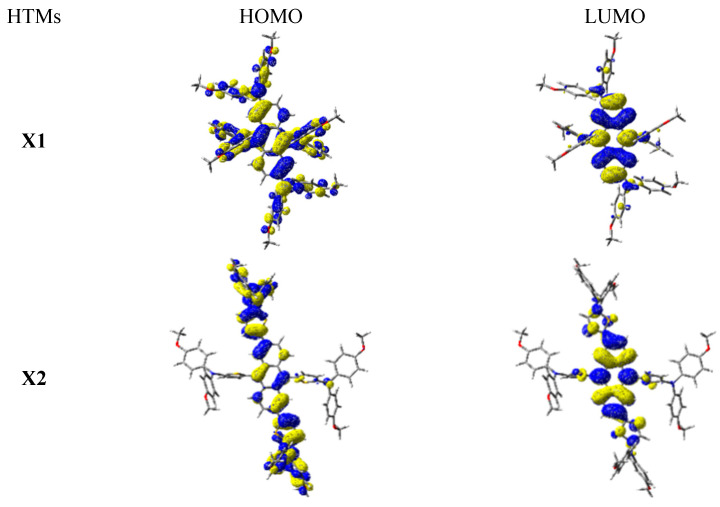
Frontier molecular orbitals for **X1** and **X2**.

To further understand the structure–property relationships of the **X1** and **X2** HTMs, theoretical calculations based on density functional theory (DFT) were conducted using the Gaussian 16 program at the B3LYP/6-31G* level. The optimized geometries and corresponding frontier molecular orbitals for **X1** and **X2** are shown in Figures S4 and S5. The dihedral angle between anthracene and thiophene at the 2,6 position ranges from 24.5° to 27.5° in **X2**, which is more planar than **X1**. Figure 3 shows that for both **X1** and **X2**, there exists significant orbital overlap between the HOMO and LUMO, mainly at the anthracene core. This clearly explains the extensive absorption exhibited by the X-type anthracene derivatives. The results also demonstrate that the two HTMs have a capacity for photo-induced electron transfer by HOMO–LUMO excitation (Figure S5). Figure S2 and Table S1 further display the calculated time-dependent DFT results and simulated UV–Vis absorption spectra, respectively. Figure S2 shows the calculated λ_max_ values of **X1** and **X2**, which are 565 nm and 551 nm, respectively. The results confirm that both compounds have low excitation energies with high oscillator strengths for the lowest-energy (S_0_→S_1_) vertical excitations, which also agrees with the experimental observations.

### 3.6. Hole-Transporting Properties

**Figure 4:**
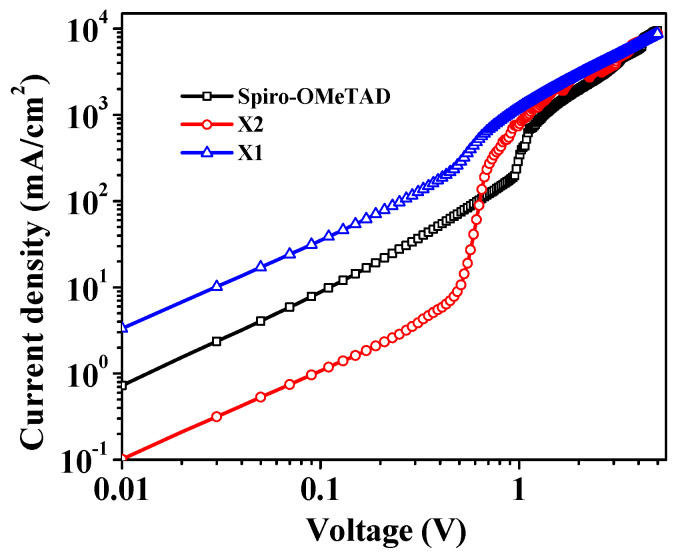
Space-charge limitation of current in the *J*−*V* characteristics of the device with **X1**, **X2**, and Spiro-OMeTAD HTMs.

To assess the charge-carrier mobility of the new synthesized molecules and spiro-OMeTAD, the ITO/PEDOT: PSS/HTM/Ag devices without additives were fabricated and recorded using the space-charge-limited-current method for the *J–V* characteristics, as shown in Figure 4. The hole mobilities of **X1**, **X2**, and spiro-OMeTAD as evaluated by Mott–Gurney were 2.8 × 10^−4^, 3.1 × 10^−4^, and 3.8 × 10^−4^ cm^2^ V^−1^ s^−1^, respectively. The higher hole-mobility value of **X2** than **X1** is due to the more planar structure between the thiophene ring and anthracene. Contrasted with **X1**, **X2** has a more planar structure at the 2,6 position of anthracene (Figure S4), which enhances the π-π stacking and facilitates the charge-transfer process. Recently, the effect of molecular planarity on the hole-mobility property has also been reported [69,70]. It was shown that the introduction of thiophene bridges can planarize the molecular structure, thereby enhancing the hole mobility. The joining of the thiophene unit in HTMs is thought of as advantageous for the imperfection passivation at the HTM/perovskite interface through the Pb-S interaction. Furthermore, the utilization of the thiophene unit can increase the level of charge delocalization and the mobility of the HTM molecule. The somewhat more ideal hole mobility could be attributed to the longer conjugation length of **X2**, which enhanced the intramolecular charge transport. Besides, the created compounds show comparable hole mobilities with spiro-OMeTAD, which might be useful to improve the performance of PSCs. A high hole mobility can efficiently conduct photo-generated holes and alleviate charge recombination.

### 3.7. Steady-State Photoluminescence (PL)

**Figure 5:**
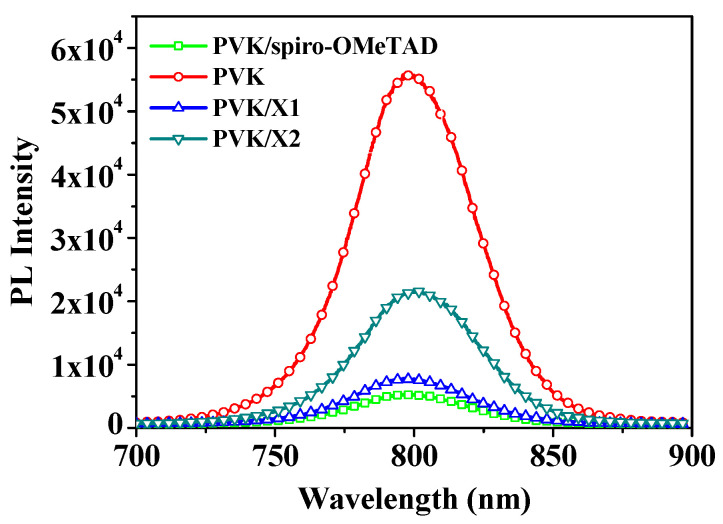
The steady-state PL spectra of perovskite with and without HTMs.

To evaluate the hole-extraction capability of new HTMs, we fabricated bilayer devices (glass/perovskite/HTM) by spin coating **X1** or **X2** on state-of-the-art mixed-cation-based perovskite films and measured their steady-state photoluminescence (PL) spectra with bare perovskite thin film as a control. Figure 5 showed the steady-state photoluminescence spectra of perovskite with and without HTMs. Pure perovskite film without the HTM layer showed a strong emission and the PL signal was fundamentally reduced, which is due to quenching the photo-generated charge carriers when the HTM was coated on the perovskite layer. **X1** exhibited high efficiency in quenching the excited perovskite when compared with **X2**, which could be attributed to its unsuitable alignment of the HOMO level for the maximum valence band of the perovskite. The result indicates that the hole transfer of **X1** with higher quenching efficiency is more efficient than that of **X2** and is also beneficial for PSC performance. In addition, spiro-OMeTAD is the most effective at quenching the excited perovskite among the three HTMs.

### 3.8. Morphology and Water-Resisting Capability

**Figure 6:**
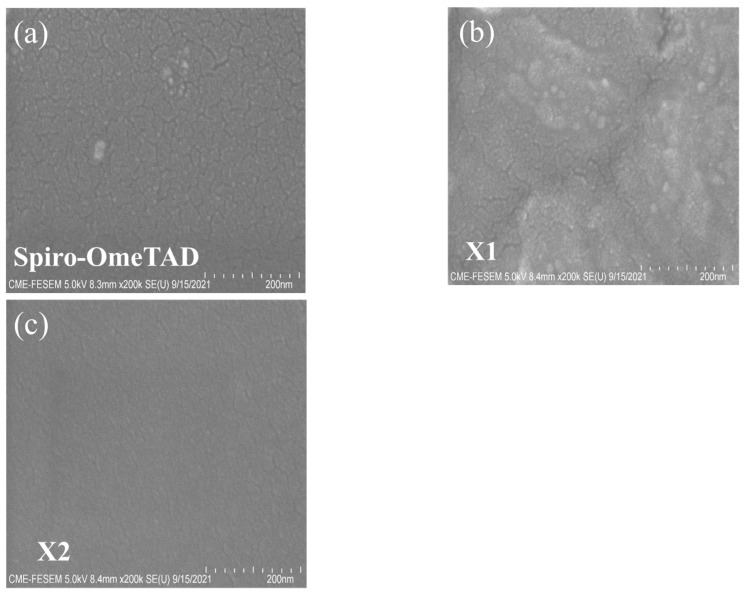
SEM images of (**a**) spiro-OMeTAD; (**b**) **X1**; and (**c**) **X2** on perovskite film.

**Figure 7:**
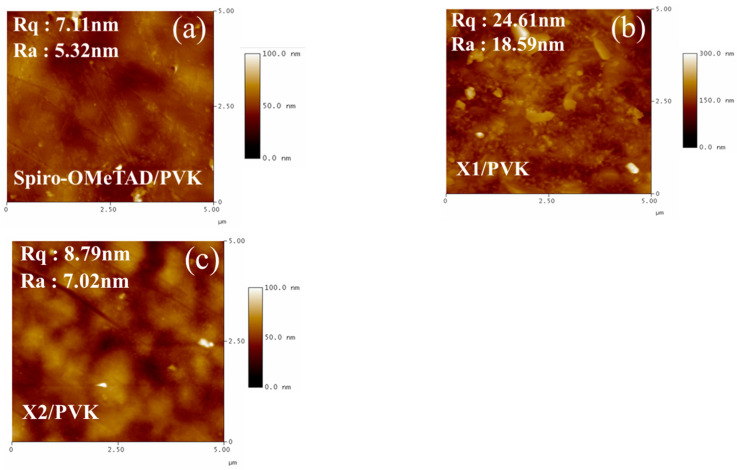
AFM images of (**a**) spiro-OMeTAD; (**b**) **X1**; and (**c**) **X2** on perovskite film.

The film-formation properties and film morphologies of **X1**, **X2**, and spiro-OMeTAD were investigated using a scanning electron microscope (SEM) and atomic force microscopy (AFM). As Figure 6a–c present, the scanning electron microscope (SEM) images were executed and the surface morphology of the HTM layer was investigated. The SEM images of **X2** and spiro-OMeTAD surfaces revealed smooth and good coverage on top of the perovskite layer, while some dots could be observed in the film of **X1**. Pinholes and particles on HTM film are ineffective at protecting the perovskite from water attacks and may adsorb more water from the air. Furthermore, pinholes may lead to a loss of the charge transporter in the device due to the direct contact between the perovskite and metal electrodes. The compact and uniform HTM film can restrict the direct contact of perovskite with Au, moisture, and oxygen, which is helpful to improve the device’s PCE and stability. In general, uniform and smoother HTM films can reduce charge recombination at the interface, which is beneficial to the improvement of device performance [71]. As shown in Figure 7, the **X1** HTM yielded a relatively larger root mean square (Rq) of 24.61 nm compared to **X2** (8.79 nm) which could be due to the poor solubility of **X1** in chlorobenzene. The **X2** film exhibited a smoother morphology with the smallest root-mean-square value, which is expected to improve charge transport in the film and across process at the perovskite/hole-transport-layer interface.

HTM plays a critical part in PSC devices, which could be viewed as a barrier for preventing water from entering the perovskite layer and stopping further consumption without encapsulation. Devices dependent on FTO/perovskite/HTM (doped) structures were utilized in the contact-angle testing. As displayed in Figure S6, the contact angles were 46.2°, 84.2°, and 73.5° for **X1**, **X2**, and spiro-OMeTAD, respectively. The hydrophobicity was decreased in the order of **X2** > spiro-OMeTAD > **X1**. This indicates that inserting the thienyl group into the molecular structure led to better hydrophobicity of the **X2** compared with the others, which might be because of its higher crystallinity and strong intermolecular interactions with the perovskite layer, which prompts unrivaled stability [72].

### 3.9. Application as HTMs in Perovskite Solar Cells

**Figure 8:**
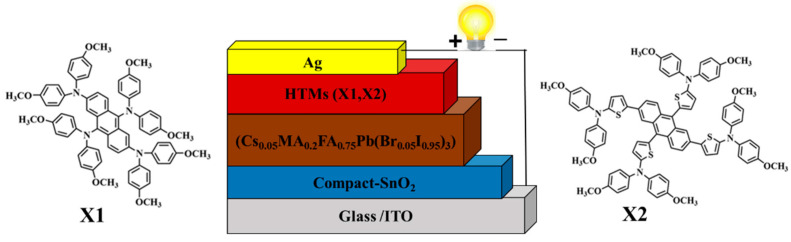
Device architecture ITO/compact SnO_2_/perovskite (Cs_0.05_MA_0.2_FA_0.75_Pb(Br_0.05_I_0.95_)_3_)/HTM (**X1**, **X2**)/Ag.

**Figure 9:**
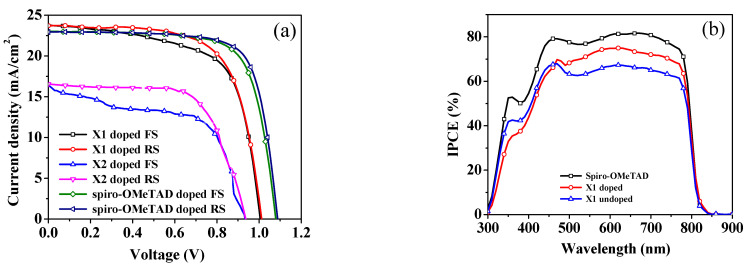

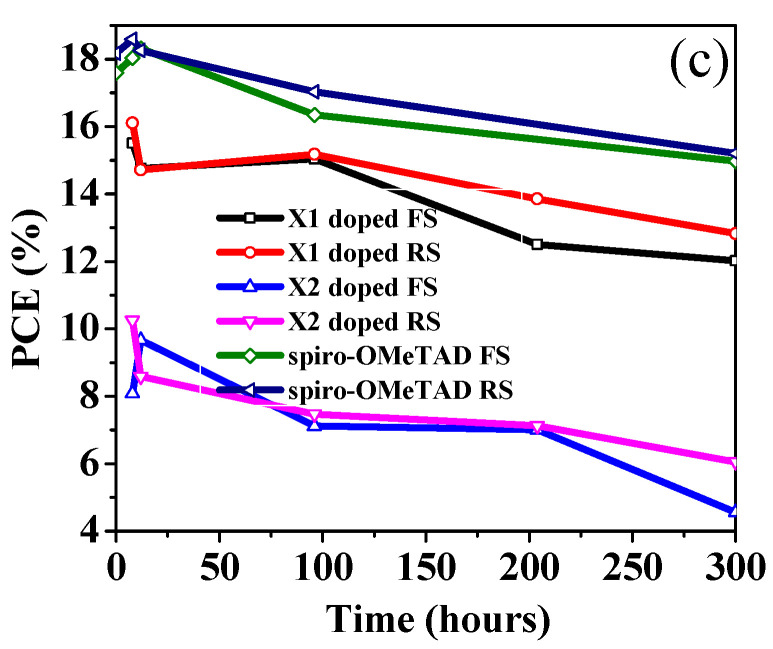
(**a**) *J*−*V* curves of the most efficient PSCs employing **X1**, **X2**, and spiro-OMeTAD under AM 1.5 illumination; (**b**) IPCE spectra for the devices with doped and undoped **X1**, and spiro-OMeTAD; (**c**) Stability test on PSCs based on the doped **X1**, **X2** and spiro-OMeTAD HTMs.

To evaluate the performances of two small-molecules HTLs, mesoporous PSC devices were fabricated comprising ITO/compact SnO_2_/MAPbI_3_/HTM/Ag, and the device design is shown in Figure 8. To appropriately evaluate the photovoltaic properties, devices containing spiro-OMeTAD as an HTM were utilized as a reference. The HTMs were applied on top of the perovskite film by spin coating from chlorobenzene solutions. The current-density–voltage (*J–V*) curves of all PSC devices, both with and without doped **X1**, **X2**, and spiro-OMeTAD, were measured under simulated solar illumination (AM 1.5G, 100 mW cm^−2^) and are shown in Figure 9 and Figure S7, respectively. The corresponding device parameters are summarized in Table 2. The **X1**-based PSC without dopants exhibited a PCE of 9.43% with a *J_SC_* of 22.228 mA cm^−2^, a *V_OC_* of 0.909 V, and an *FF* of 47.00%. The **X2**-based PSC without dopants showed extremely lower efficiency with a PCE of 0.598%. When **X1** and **X2** were doped with TPB and Li-TFSI as additives, the PSC performance significantly improved. Figure 9a shows the best PSC, which was based on the doped **X1**, yielded a PCE of 16.10%, a *J_SC_* of 23.72 mA cm^−2^, a *V_OC_* of 1.01 V, and an *FF* of 67.54%, which is obviously higher than the doped **X2** (PCE = 10.26%, *J_SC_* = 16.57 mA cm^−2^, *V_OC_* = 0.94 V, *FF* = 66.17%). The PSC device utilizing **X1** showed the best performance regardless of doping. The higher *V_OC_* for the **X1**-based PSC may be due to its lower HOMO energy level. The device based on **X1** showed a preferable photovoltaic parameter compared to the **X2**-based device, which can be related to its better hole-extraction ability, as discussed in the section on steady-state PL, and close to that of spiro-OMeTAD as an HTM, which showed a *J_SC_* of 22.93 mA cm^−2^, a *V_OC_* of 1.09 V, an *FF* of 75.01, and a PCE of 18.58% under the same conditions. From SEM and AFM images, the poor film-forming properties of **X1** and **X2** may lead to inferior charge-extraction capability, as illustrated in PL quenching measurements. It should be noted that the FF value of the spiro-OMeTAD-based PSC being higher than both of the HTMs may be due to the smoother morphology when spin coating on the surface of the perovskite layer. Figure 9b shows the incident-photon-to-current-conversion-efficiency (IPCE) spectra of the most efficient devices that were based on **X1** as the HTM, with and without doping. In comparison with the spiro-OMeTAD-based cell, the IPCE values of both the doped and undoped **X1**-based devices were lower, in the range from 350 to 800 nm. It can be seen that the IPCE responses in the 450 to 750 nm range were higher than 60% for both the doped and undoped **X1** devices. The long-term stability is very important for PSCs. Therefore, we performed an aging test on the most efficient cell devices with doped **X1** and compared it with that of spiro-OMeTAD. Table S2 summarized the device efficiency parameters and Figure 9c plots the attenuation curve of the PCE of these devices. The devices were stored without encapsulation with around 30% relative humidity. As shown in Figure 9c, the devices with doped **X1** maintained about 80% of their initial PCE after 300 h. In comparison, the spiro-OMeTAD-based device maintained 88% of its initial PCE during the same period. This is consistent with spiro-OMeTAD having higher hydrophobicity than **X1**, as mentioned above.

## 4. Conclusions

In summary, we successfully synthesized two simple hole-transporting materials **X1** and **X2** that consolidated anthracene as a π-linker using a facile synthesis process. Both HTMs showed similar photophysical, electrochemical, and thermal properties. A thermal analysis of the synthesized materials revealed that their thermal stability was sufficiently high for practical application in photovoltaic devices. **X2** was subjected to insertion of a thiophene between the anthracene and diarylamine groups, resulting in an upward shift of the HOMO level, high hole mobility, and hindering water permeation compared with **X1**. From the SEM and AFM images, it was shown that the films of **X1** and **X2** were not smoother than those of spiro-OMeTAD. Moreover, steady-state PL experiments exhibited better hole extraction and transfer in the case of **X1**. Subsequently, the PSC fabricated with doped **X1** as the HTM showed a PCE of 16.10%, outperforming the devices that were fabricated utilizing the other HTM **X2** (10.25%) under simulated solar illumination (AM 1.5G, 100 mW cm^−2^). The HOMO levels of the **X1** and **X2** HTMs were estimated to be higher than that of spiro-OMeTAD, which led to lower measured V_OC_ values. The efficiency of the unencapsulated devices based on the **X1** HTM could be maintained at 80% of the initial cell efficiency after 300 h under ambient conditions. We believe that this study provides the molecular-design strategy for developing efficient HTMs based on *2*,*6*,*9*,*10*-substituted anthracene for stable PSCs and future applications.

## Figures and Tables

**Table 1:** Optical, cyclic-voltammetry, thermal, hole-mobility properties of **X1**, **X2**, spiro-OMeTAD.

HTM	λ_max_ ^a^ (Soln) (ε × 10^−5^ M^−1^ cm^−1^) (nm)	λ_max_^b^ (Film) (nm)	λ_em_ ^a^ (Soln) (nm)	E_0–0_ ^c^ (ev)	HOMO/ LUMO (eV)	T_d_/T_g_ ^d^ (°C)	µ_h_^e^ (cm^2^ V^−1^ s^−1^)
**X1**	524 (1.49)	540	564	2.28	−5.03/−2.76	424/135	2.8 × 10^−4^
**X2**	486 (3.12)	525	589	2.31	−4.94/−2.63	422/95	3.1 × 10^−4^
Spiro-OMeTAD [60]	385 (5.24)	396	430	3.04	−5.15/−2.11	422/129	3.8 × 10^−4^

^a^ Absorption and emission spectra were measured in dichloromethane. ^b^ Absorption spectra were recorded on CHCl_3_ film. ^c^ E_0–0_ was determined from the intersection of absorption and emission spectra in DCM. ^d^ T_d_/T_g_ was determined from TGA/DSC. ^e^ Hole mobilities of HTMs.

**Table 2:** Photovoltaic performances of PSCs with **X1** and **X2** HTMs compared with the reference HTM with spiro-OMeTAD.

HTM		Scan Direction	*V*_OC_ (V) (Average) ^a^	*J*_SC_ (mA/cm^2^) (Average) ^a^	*FF* (Average) ^a^	PCE (%) Best	PCE (%) (Average) ^a^
**X1**	Undoped	FS	0.909 (0.882 ± 0.040)	22.228 (21.752 ± 0.379)	47.006 (42.445 ± 3.012)	9.43	8.098 ± 0.827
	Undoped	RS	0.911 (0.900 ± 0.011)	22.325 (21.883 ± 0.297)	45.804 (40.643 ± 3.458)	9.25	7.966 ± 0.825
	Doped	FS	1.005 (0.941 ± 0.113)	23.799 (22.147 ± 1.609)	65.269 (62.612 ± 6.389)	15.51	12.926 ± 2.091
	Doped	RS	1.012 (0.974 ± 0.019)	23.721 (22.714 ± 0.705)	67.543 (62.744 ± 4.351)	16.10	13.793 ± 1.209
**X2**	Undoped	FS	0.303 (0.152 ± 0.145)	5.523 (4.332 ± 0.859)	35.943 (18.622 ± 6.842)	0.59	0.279 ± 0.281
	Undoped	RS	0.029 (0.018 ± 0.006)	4.837 (4.095 ± 0.371)	24.156 (22.161 ± 5.711)	0.03	0.018 ± 0.009
	Doped	FS	0.933 (0.901 ± 0.534)	16.432 (14.137 ± 1.109)	52.763 (55.412 ± 1.878)	8.08	7.005 ± 1.659
	Doped	RS	0.936 (0.831 ± 0.676)	16.568 (14.842 ± 1.223)	66.165 (58.326 ± 1.229)	10.25	7.126 ± 1.407
Spiro-OMeTAD	Doped	FS	1.079 (1.066 ± 0.014)	23.022 (22.646 ± 0.401)	73.056 (71.189 ± 1.502)	18.03	17.001 ± 0.603
	Doped	RS	1.088 (1.074 ± 0.012)	22.934 (22.608 ± 0.371)	75.012 (72.298 ± 1.747)	18.58	17.104 ± 0.726

^a^ The average photovoltaic values were estimated based on 10 PSC devices.

## Data Availability

The data presented in this study are available on request from the corresponding author.

## Supplementary Materials

The following are available online at https://www.mdpi.com/article/10.3390/polym14081580/s1, Figure S1: Absorption and emission spectra of **X1**, **X2** recorded in different solvents. Figure S2: Simulated UV–Vis absorption spectra of **X1**, **X2** at TD-DFT/CPCM/CAM-B3LYP/6-31g*, gas phase. Figure S3: (a) Thermogravimetric analysis of **X1**, **X2** at a scan rate of 10 °C min^−1^ under N_2_ atmosphere. (b) Differential scanning calorimetry of **X1**, **X2** under nitrogen at a heating rate of 20 °C min^−1^. Figure S4: Calculated molecular geometries and dihedral angles between adjacent groups for **X1** and **X2**. Figure S5: Frontier molecular orbitals of electron distributions in HOMO and LUMO levels for **X1** and **X2** respectively. Figure S6: Contact angle between water and HTM film of **X1**, **X2**, and spiro-OMeTAD on perovskite. Figure S7: *J*–*V* curves of PSCs undoped **X1**, **X2** under forward and reverse scan measured under AM 1.5G illuminations in ambient atmosphere at a constant rate 100 mV s^−1^. Figure S8: ^1^H NMR spectrum of **X1**. Figure S9: ^13^C NMR spectrum of **X1**. Figure S10: ^1^H NMR spectrum of **X2**. Figure S11: ^13^C NMR spectrum of **X2**. Table S1: Lowest-energy singlet excited states calculated at the TDDFT B3LYP/6-31G* level for in Gas Phase. Vertical excitation energies (E), oscillator strengths (f) and dominant monoexcitations with contributions (within parentheses) greater than 10%. Table S2: Device stability parameters of perovskite solar cells based on the **X1**, **X2**, Spiro-OMeTAD HTMs.

## References

[1] Kojima A., Teshima K., Shirai Y., Miyasaka T. (2009). Organometal Halide Perovskites as Visible-Light Sensitizers for Photovoltaic Cells. J. Am. Chem. Soc..

[2] Ding X., Wang H., Chen C., Li H., Tian Y., Li Q., Cheng M. (2021). Passivation functionalized phenothiazine-based hole transport material for highly efficient perovskite solar cell with efficiency exceeding 22%. Chem. Eng. J..

[3] Sathiyan G., Sivakumar E.K.T., Ganesamoorthy R., Thangamuthu R., Sakthivel P. (2016). Review of carbazole based conjugated molecules for a highly efficient organic solar cell application. Tetrahedron Lett..

[4] Pham H.D., Xianqiang L., Li W., Manzhos S., Kyaw A.K.K., Sonar P. (2019). Organic interfacial materials for perovskite-based optoelectronic devices. Energy Environ. Sci..

[5] Zhang J., Morbidoni M., Huang K., Feng S., McLachlan M.A. (2018). Environmentally friendly, aqueous processed ZnO as an efficient electron transport layer for low temperature processed metal–halide perovskite photovoltaics. Inorg. Chem. Front..

[6] Calió L., Kazim S., Grätzel M., Ahmad S. (2016). Hole-transport materials for perovskite solar cells. Angew. Chem. Int. Ed..

[7] Duan L., Chen Y., Zong X., Liu R., Sun Z., Liang M., Xue S. (2019). Facile synthesis of triphenylamine-based hole-transporting materials for planar perovskite solar cells. J. Power Sources.

[8] Zhao L., Qiu L., Xia D., Liu S., Yi X., Fan J., Yang Y. (2019). Cyclooctatetrathiophene-Cored Three-Dimensional Hole Transport Material Enabling Over 19% Efficiency of Perovskite Solar Cells. ACS Appl. Energy Mater..

[9] Liu C., Zhang D., Li Z., Zhang X., Shen L., Guo W. (2018). Efficient *4*, *4*′, *4*″-tris (*3*-methylphenylphenylamino) triphenylamine (m-MTDATA) Hole Transport Layer in Perovskite Solar Cells Enabled by Using the Nonstoichiometric Precursors. Adv. Funct. Mater..

[10] Bai L., Wang Z., Han Y., Zuo Z., Liu B., Yu M., Huang W. (2018). Diarylfluorene-based nano-molecules as dopant-free hole-transporting materials without post-treatment process for flexible pin type perovskite solar cells. Nano Energy.

[11] Kung P.K., Li M.H., Lin P.Y., Chiang Y.H., Chan C.R., Guo T.F., Chen P. (2018). A review of inorganic hole transport materials for perovskite solar cells. Adv. Mater. Interfaces.

[12] Qin P., Paek S., Dar M.I., Pellet N., Ko J., Grätzel M., Nazeeruddin M.K. (2014). Perovskite solar cells with 12.8% efficiency by using conjugated quinolizino acridine based hole transporting material. J. Am. Chem. Soc..

[13] Li H., Fu K., Hagfeldt A., Grätzel M., Mhaisalkar S.G., Grimsdale A.C. (2014). A simple *3*,*4*-ethylenedioxythiophene based hole-transporting material for perovskite solar cells. Angew. Chem..

[14] Krishna A., Sabba D., Li H., Yin J., Boix P.P., Soci C., Grimsdale A.C. (2014). Novel hole transporting materials based on triptycene core for high efficiency mesoscopic perovskite solar cells. Chem. Sci..

[15] Christians J.A., Fung R.C., Kamat P.V. (2014). An inorganic hole conductor for organo-lead halide perovskite solar cells. Improved hole conductivity with copper iodide. J. Am. Chem. Soc..

[16] Chavhan S., Miguel O., Grande H.J., Gonzalez-Pedro V., Sánchez R.S., Barea E.M., Tena-Zaera R. (2014). Organo-metal halide perovskite-based solar cells with CuSCN as the inorganic hole selective contact. J. Mater. Chem. A.

[17] Cheng M., Chen C., Yang X., Huang J., Zhang F., Xu B., Sun L. (2015). Novel small molecular materials based on phenoxazine core unit for efficient bulk heterojunction organic solar cells and perovskite solar cells. Chem. Mater..

[18] Chen H., Bryant D., Troughton J., Kirkus M., Neophytou M., Miao X., McCulloch I. (2016). One-step facile synthesis of a simple hole transport material for efficient perovskite solar cells. Chem. Mater..

[19] Saliba M., Orlandi S., Matsui T., Aghazada S., Cavazzini M., Correa-Baena J.P., Nazeeruddin M.K. (2016). A molecularly engineered hole-transporting material for efficient perovskite solar cells. Nat. Energy.

[20] Xu B., Zhang J., Hua Y., Liu P., Wang L., Ruan C., Sun L. (2017). Tailor-making low-cost spiro [fluorene-*9*,*9*′-xanthene]-based 3D oligomers for perovskite solar cells. Chem.

[21] Ge Q.Q., Shao J.Y., Ding J., Deng L.Y., Zhou W.K., Chen Y.X., Zhong Y.W. (2018). A Two-Dimensional Hole-Transporting Material for High-Performance Perovskite Solar Cells with 20% Average Efficiency. Angew. Chem. Int. Ed..

[22] Chiykowski V.A., Cao Y., Tan H., Tabor D.P., Sargent E.H., Aspuru-Guzik A., Berlinguette C.P. (2018). Precise Control of Thermal and Redox Properties of Organic Hole-Transport Materials. Angew. Chem. Int. Ed..

[23] Christians J.A., Schulz P., Tinkham J.S., Schloemer T.H., Harvey S.P., de Villers B.J.T., Luther J.M. (2018). Tailored interfaces of unencapsulated perovskite solar cells for >1000 hour operational stability. Nat. Energy.

[24] Vaitukaityte D., Wang Z., Malinauskas T., Magomedov A., Bubniene G., Jankauskas V., Snaith H.J. (2018). Efficient and Stable Perovskite Solar Cells Using Low-Cost Aniline-Based Enamine Hole-Transporting Materials. Adv. Mater..

[25] Zhu X.D., Ma X.J., Wang Y.K., Li Y., Gao C.H., Wang Z.K., Liao L.S. (2019). Hole-Transporting Materials Incorporating Carbazole into Spiro-Core for Highly Efficient Perovskite Solar Cells. Adv. Funct. Mater..

[26] Shen C., Wu Y., Zhang H., Li E., Zhang W., Xu X., Zhu W.H. (2019). Semi-Locked Tetrathienylethene as a Building Block for Hole-Transporting Materials: Toward Efficient and Stable Perovskite Solar Cells. Angew. Chem. Int. Ed..

[27] Heo J.H., Park S., Im S.H., Son H.J. (2017). Development of Dopant-Free Donor−Acceptor-type Hole Transporting Material for Highly Efficient and Stable Perovskite Solar Cells. ACS Appl. Mater. Interfaces.

[28] Li Y., Scheel K.R., Clevenger R.G., Shou W., Pan H., Kilway K.V., Peng Z. (2018). Highly Efficient and Stable Perovskite Solar Cells Using a Dopant-Free Inexpensive Small Molecule as the Hole-Transporting Material. Adv. Energy Mater..

[29] Jiang X., Wang D., Yu Z., Ma W., Li H.B., Yang X., Liu F., Hagfeldt A., Sun L. (2019). Molecular Engineering of Copper Phthalocyanines: A Strategy in Developing Dopant-Free Hole-Transporting Materials for Efficient and Ambient-Stable Perovskite Solar Cells. Adv. Energy Mater..

[30] Molina-Ontoria A., Zimmermann I., Garcia-Benito I., Gratia P., Roldán-Carmona C., Aghazada S., Martín N. (2016). Benzotrithiophene-Based Hole-Transporting Materials for 18.2% Perovskite Solar Cells. Angew. Chem..

[31] Schwarzburg K., Willig F. (2003). Diffusion impedance and space charge capacitance in the nanoporous dye-sensitized electrochemical solar cell. J. Phys. Chem. B.

[32] Nishimura H., Ishida N., Shimazaki A., Wakamiya A., Saeki A., Scott L.T., Murata Y. (2015). Hole-transporting materials with a two-dimensionally expanded π-system around an azulene core for efficient perovskite solar cells. J. Am. Chem. Soc..

[33] Teh C.H., Daik R., Lim E.L., Yap C.C., Ibrahim M.A., Ludin N.A., Teridi M.A.M. (2016). A review of organic small molecule-based hole-transporting materials for meso-structured organic–inorganic perovskite solar cells. J. Mater. Chem. A.

[34] Liu X., Kong F., Cheng T., Chen W., Tan Z.A., Yu T., Dai S. (2017). Tetraphenylmethane-arylamine hole-transporting materials for perovskite solar cells. ChemSusChem.

[35] Zhang X., Liu X., Ghadari R., Li M., Zhou Z.A., Ding Y., Dai S. (2021). Tetraphenylethylene-Arylamine Derivatives as Hole Transporting Materials for Perovskite Solar Cells. ACS Appl. Mater. Interfaces.

[36] Chen Y.C., Yen J.H., Chung C.L., Chen C.P. (2019). Methoxy groups on bifluorenylidene-based hole transporting materials result in highly efficient and stable dopant-free inverted perovskite solar cells. Sol. Energy.

[37] Urieta-Mora J., Garcia-Benito I., Zimmermann I., Arago J., Molina-Ontoria A., Orti E., Nazeeruddin M.K. (2019). Tetrasubstituted thieno [*3*,*2*-*b*] thiophenes as hole-transporting materials for perovskite solar cells. J. Org. Chem..

[38] Lai K.W., Chang C.C., Chu C.W. (2021). Benzodithiophene-based small molecules with various termini as hole transporting materials in efficient planar perovskite solar cells. Org. Electron..

[39] Cho A.N., Chakravarthi N., Kranthiraja K., Reddy S.S., Kim H.S., Jin S.H., Park N.G. (2017). Acridine-based novel hole transporting material for high efficiency perovskite solar cells. J. Mater. Chem. A.

[40] Yao H., Wu T., Wu B., Zhang H., Wang Z., Sun Z., Liang M. (2021). The triple π-bridge strategy for tailoring indeno [2,1-*b*] carbazole-based HTMs enables perovskite solar cells with efficiency exceeding 21%. J. Mater. Chem. A.

[41] Yin X., Zhou J., Song Z., Dong Z., Bao Q., Shrestha N., Tang W. (2019). Dithieno [*3*, *2*-*b*:*2*′, *3*′-*d*] pyrrol-Cored hole transport material enabling over 21% efficiency dopant-free perovskite solar cells. Adv. Funct. Mater..

[42] Guo H., Zhang H., Shen C., Zhang D., Liu S., Wu Y., Zhu W.H. (2021). A coplanar π-extended quinoxaline based hole-transporting material enabling over 21% efficiency for dopant-free perovskite solar cells. Angew. Chem. Int. Ed..

[43] Cheng Y., Fu Q., Zong X., Dong Y., Zhang W., Wu Q., Xue S. (2021). Coplanar phenanthro [*9*, *10*-*d*] imidazole based hole-transporting material enabling over 19%/21% efficiency in inverted/regular perovskite solar cells. Chem. Eng. J..

[44] Akin S., Bauer M., Uchida R., Arora N., Jacopin G., Liu Y. (2020). Cyclopentadithiophene-based hole-transporting material for highly stable perovskite solar cells with stabilized efficiencies approaching 21%. ACS Appl. Energy Mater..

[45] Deng Z., He M., Zhang Y., Ullah F., Ding K., Liang J., Chen C.C. (2020). Design of Low Crystallinity Spiro-Typed Hole Transporting Material for Planar Perovskite Solar Cells to Achieve 21.76% Efficiency. Chem. Mater..

[46] Park J.H., Chung D.S., Lee D.H., Kong H., Jung I.H., Park M.J., Cho N.S. (2010). New anthracene thiophene-based copolymers that absorb across the entire UV-vis spectrum for application in organic solar cells. Chem. Commun..

[47] Ma J.Y., Yun H.J., Kim S.O., Lee G.B., Cha H.J., Park C.E., Kwon S.K., Kim Y.H. (2014). Novel Alkoxyanthracene Donor and Benzothiadiazole Acceptor for Organic Thin Film Transistor and Bulk Heterojunction Organic Photovoltaic Cells. J. Polym. Sci. Part A Polym. Chem..

[48] Shih P.-I., Chuang C.-Y., Chien C.-H., Diau E.W.-G., Shu C.-F. (2007). Highly efficient non-doped blue-light-emitting diodes based on an anthracene derivative end capped with tetraphenylethylene groups. Adv. Funct. Mater..

[49] Wang J., Wan W., Jiang H., Gao Y., Jiang X., Lin H., Zhao W., Hao J. (2010). C-9 Fluorenyl substituted anthracenes: A promising new family of blue luminescent materials. Org. Lett..

[50] Wee K.-R., Han W.-S., Kim J.-E., Kim A.-L., Kwon S., Kang S.O. (2011). Asymmetric anthracene-based blue host materials: Synthesis and electroluminescence properties of *9*-(*2*-naphthyl)-*10*-arylanthracenes. J. Mater. Chem..

[51] Chung D.S., Park J.W., Park J.H., Moon D., Kim G.H., Lee D.H., Shim H.K., Kwon S.K., Park C.E. (2010). High mobility organic single crystal transistors based on soluble triisopropylsilylethynyl anthracene derivatives. J. Mater. Chem..

[52] Jung K.H., Bae S.Y., Kim K.H., Cho M.J., Lee K., Kim Z.H., Choi D.H., Chung D.S., Park C.E. (2009). High-mobility anthracene-based X-shaped conjugated molecules for thin film transistors. Chem. Commun..

[53] Teng C., Yang X., Yang C., Li S., Cheng M., Hagfeldt A., Sun L. (2010). Molecular design of anthracene-bridged metal-free organic dyes for efficient dye-sensitized solar cells. J. Phys. Chem. C.

[54] Thomas K.R.J., Singh P., Baheti A., Hsu Y.-C., Ho K.-C., Lin J.T. (2011). Electro-optical properties of new anthracene based organic dyes for dye-sensitized solar cells. Dyes Pigments.

[55] Marrocchi A., Silvestri F., Seri M., Facchetti A., Taticchi A., Marks T.J. (2009). Conjugated anthracene derivatives as donor materials for bulk heterojunction solar cells: Olefinic versus acetylenic spacers. Chem. Commun..

[56] Liu X., Kong F., Ghadari R., Jin S., Yu T., Chen W., Dai S. (2017). Anthracene–arylamine hole transporting materials for perovskite solar cells. Chem. Commun..

[57] Pham H.D., Hu H., Wong F.L., Lee C.S., Chen W.C., Feron K., Sonar P. (2018). Acene-based organic semiconductors for organic light-emitting diodes and perovskite solar cells. J. Mater. Chem. C.

[58] Singh A., Abate S.Y., Pavan Kumar C., Wu W.T., Hsiao J.C., Wu F.L., Tao Y.T. (2020). Bis (diphenylamine)-Tethered Carbazolyl Anthracene Derivatives as Hole-Transporting Materials for Stable and High-Performance Perovskite Solar Cells. ACS Appl. Energy Mater..

[59] Liu C.C., Cai W.Z., Guan X., Duan C.H., Xue Q.F., Ying L., Huang F., Cao Y. (2013). Synthesis of donor-acceptor copolymers based on anthracene derivatives for polymer solar cells. Polym. Chem..

[60] Chandrasekaran D., Chiu Y.L., Yu C.K., Yen Y.S., Chang Y.J. (2021). Polycyclic Arenes Dihydrodinaphthopentacene-based Hole-Transporting Materials for Perovskite Solar Cells Application. Chem. Asian J..

[61] Hsu C.-P. (2009). The Electronic Couplings in Electron Transfer and Excitation Energy Transfer. Acc. Chem. Res..

[62] Dang D., Zhou P., Wu Y., Xu Y., Zhi Y., Zhu W. (2018). Isomeric Organic Semiconductors Containing Fused-Thiophene Cores: Molecular Packing and Charge Transport. Phys. Chem. Chem. Phys..

[63] Del Rosso P.G., Almassio M.F., Bruno M., Garay R.O. (2010). Mild persubstitution of di-and tetrabrominated arenes with arylthiolate nucleophiles. Tetrahedron Lett..

[64] Li Y., Scudiero L., Ren T., Dong W.J. (2012). Synthesis and characterizations of benzothiadiazole-based fluorophores as potential wavelength-shifting materials. J. Photochem. Photobiol. A Chem..

[65] Leliège A., Grolleau J., Allain M., Blanchard P., Demeter D., Rousseau T., Roncali J. (2013). Small D–π–A Systems with o-Phenylene-Bridged Accepting Units as Active Materials for Organic Photovoltaics. Chem. Eur. J..

[66] Maciejczyk M., Ivaturi A., Robertson N. (2016). SFX as a low-cost ‘Spiro’ hole-transport material for efficient perovskite solar cells. J. Mater. Chem. A.

[67] Grabowski Z.R., Rotkiewicz K., Rettig W. (2003). Structural Changes Accompanying Intramolecular Electron Transfer:  Focus on Twisted Intramolecular Charge-Transfer States and Structures. Chem. Rev..

[68] Hagfeldt A., Boschloo G., Sun L., Kloo L., Pettersson H. (2010). Dye-sensitized solar cells. Chem. Rev..

[69] Zhou X., Kong F., Sun Y., Huang Y., Zhang X., Ghadari R. (2020). Benzothiadiazole-based hole transport materials for high-efficiency dopant-free perovskite solar cells: Molecular Planarity effect. J. Energy Chem..

[70] Tao L., Chen C., Wu C., Ding X., Zheng M., Li H., Li G., Lu H., Cheng M. (2020). Fluorine-Substituted Benzotriazole Core Building Block-Based Highly Efficient Hole-Transporting Materials for Mesoporous Perovskite Solar Cells. Sol. RRL.

[71] Lee S., Park K.H., Lee J.H., Back H., Sung M.J., Lee J., Lee K. (2019). Achieving thickness-insensitive morphology of the photoactive layer for printable organic photovoltaic cells via side chain engineering in nonfullerene acceptors. Adv. Energy Mater..

[72] Swick S.M., Zhu W., Matta M., Aldrich T.J., Harbuzaru A., Lopez Navarrete J.T., Ponce Ortiz R., Kohlstedt K.L., Schatz G.C.A., Facchetti F.S. (2018). Closely Packed, Low Reorganization Energy π-extended Post fullerene Acceptors for Efficient Polymer Solar Cells. Proc. Natl. Acad. Sci. USA.

