# Supplementary material for: Effect of Thiophene Insertion on X-Shaped Anthracene-Based Hole-Transporting Materials in Perovskite Solar Cells
Source: Polymers (Basel). 2022 Apr 13;14(8):1580. doi: 10.3390/polym14081580 (PMC9024987; doi:10.3390/polym14081580)
Supplement: Supplementary file 1 [file polymers-14-01580-s001.zip › polymers-1659389-supplementary.pdf]

polymers

MDPI

Supplementary

Effect of Thiophene Insertion on X-Shaped Anthracene-Based Hole-Transporting Materials in Perovskite Solar Cells

Dharuman Chandrasekaran [1], Wei Hao Chiu [2], Kun Mu Lee [2,3,4,5,*], Jian Ming Liao [6], Hsien Hsin Chou [6,*] and Yung Sheng Yen [1,*]

[1] Department of Chemistry, Chung Yuan Christian University, 320 Zhongli, Taoyuan, Taiwan; chandrugood91@gmail.com
[2] Center for Reliability Sciences and Technologies, Chang Gung University, 333 Guishan, Taoyuan, Taiwan; weihao.chiu@gmail.com
[3] Department of Chemical and Materials Engineering, Chang Gung University, 333 Guishan, Taoyuan, Taiwan
[4] Center for Green Technology, Chang Gung University, 333 Guishan, Taoyuan, Taiwan
[5] Division of Neonatology, Department of Pediatrics, Chang Gung Memorial Hospital, 33305 Linkou, Taoyuan, Taiwan
[6] Department of Applied Chemistry, Providence University, 433 Taichung, Taiwan; jl387555@gmail.com
* Correspondence: kmlee@mail.cgu.edu.tw (K.M.L.); hhchou@pu.edu.tw (H.H.C.); ysyen@cycu.edu.tw (Y.S.Y.)

Figures and tables

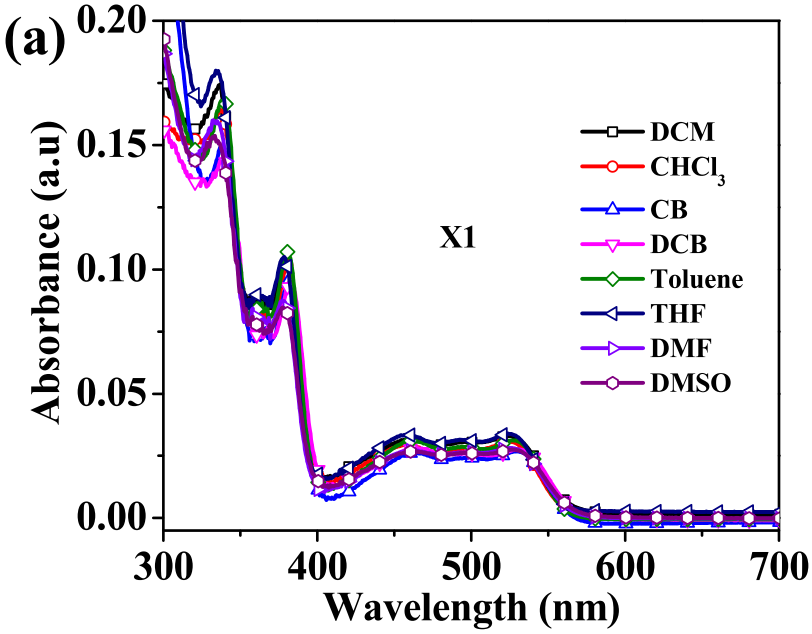

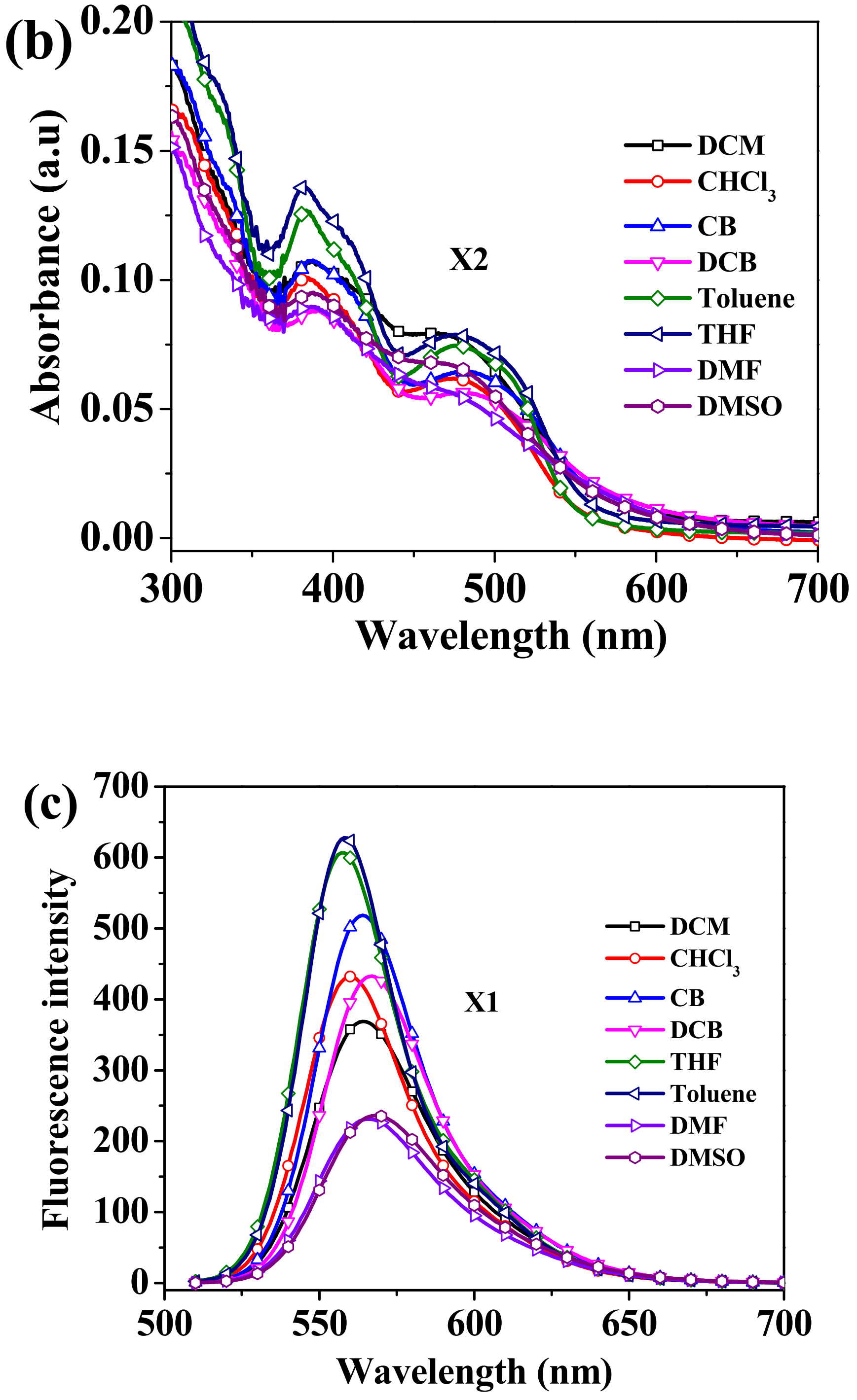
(b)
Absorbance (a.u)
0.00
0.05
0.10
0.15
0.20
300
400
500
600
700
Wavelength (nm)
X2
DCM
CHCl3
CB
DCB
Toluene
THF
DMF
DMSO
(c)
Fluorescence intensity
0
100
200
300
400
500
600
700
500
550
600
650
700
Wavelength (nm)
X1
DCM
CHCl3
CB
DCB
THF
Toluene
DMF
DMSO

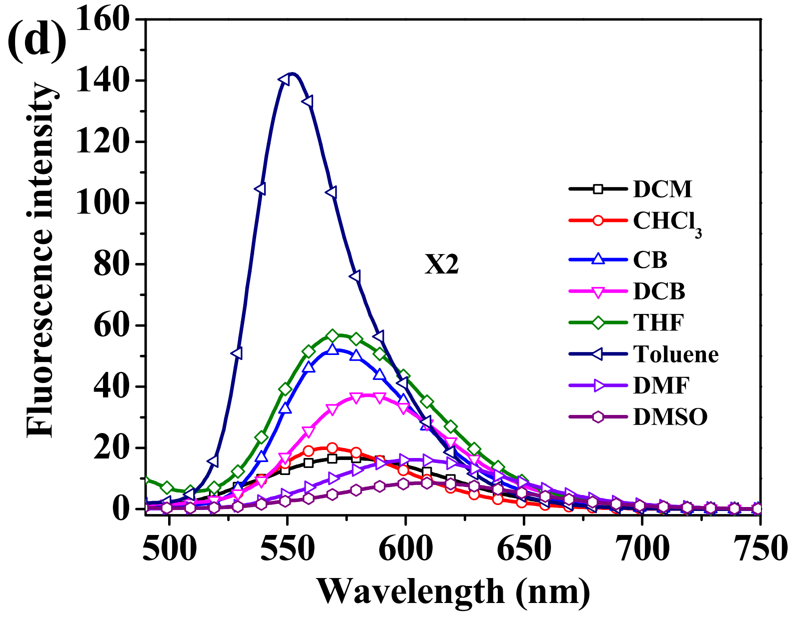

Figure S1. (**a**), (**b**) Absorption spectra of **X1**, **X2** (**c**), (**d**) Emission spectra of **X1**, **X2** recorded in different solvents.

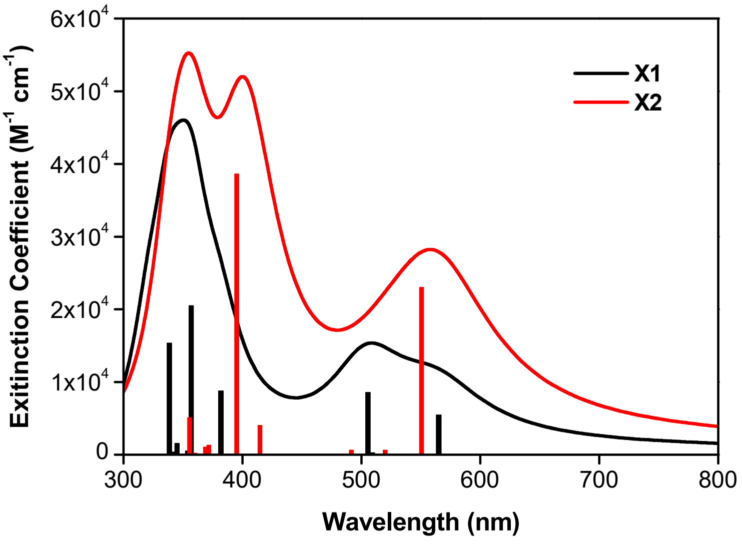

Figure S2. Simulated UV-vis absorption spectra for **X1** and **X2** based on TDDFT results.

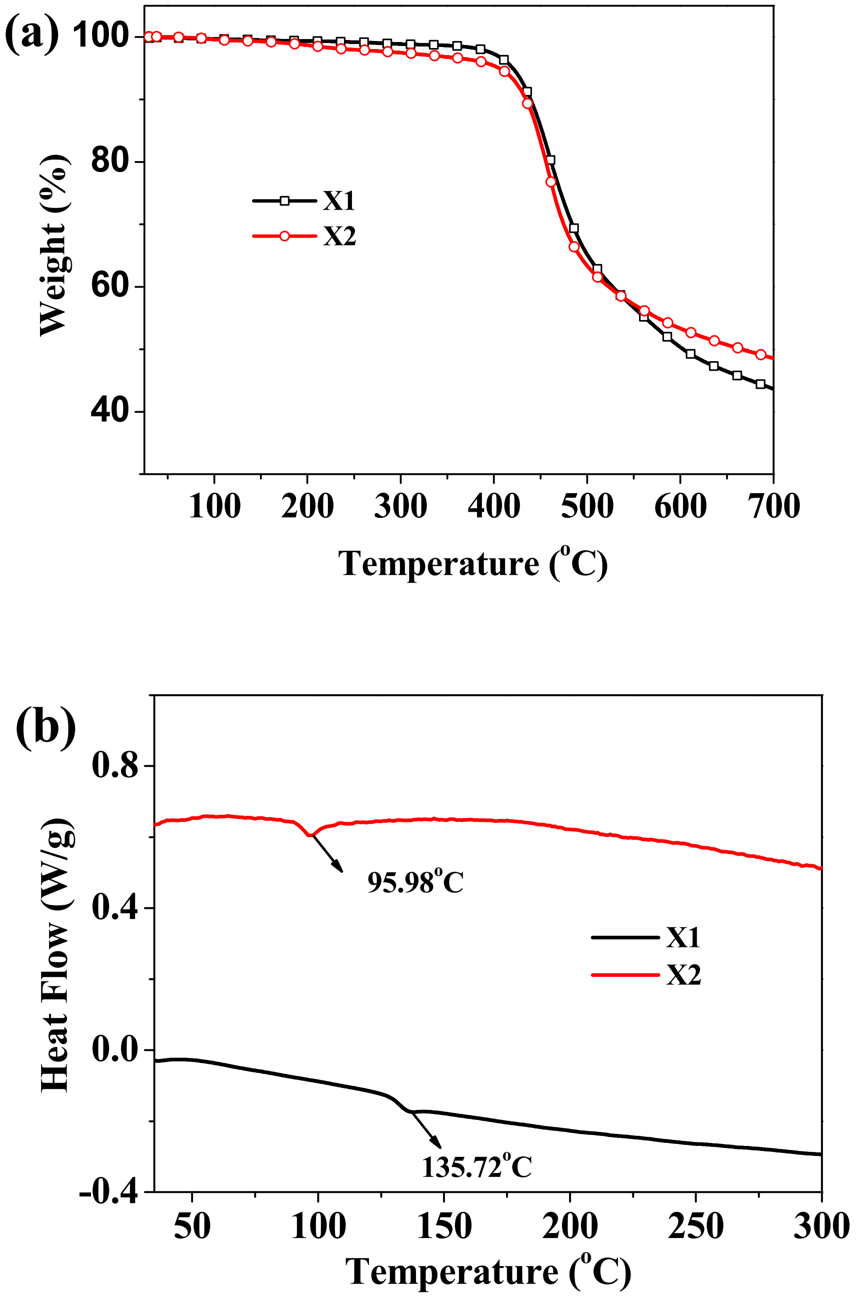

Figure S3. (**a**) Thermogravimetric analysis of **X1**, **X2** at a scan rate of 10 °C min^{-1} under N_2 atmosphere. (**b**) Differential scanning calorimetry of **X1**, **X2** under nitrogen at a heating rate of 20 °C min^{-1}.

<table>
<tr><th>HTMs</th><th>Top view</th><th>Side view</th></tr>
<tr><td>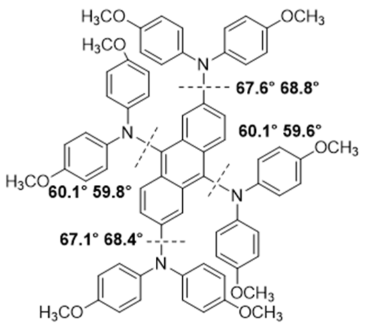

X1</td><td>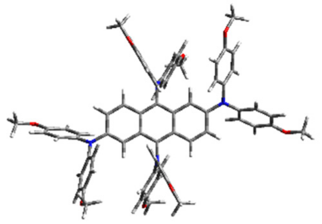</td><td>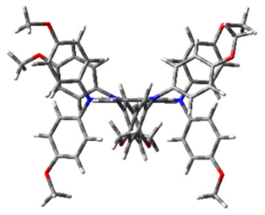</td></tr>
<tr><td>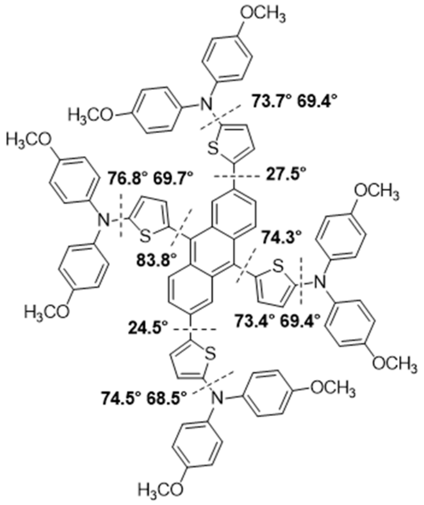

X2</td><td>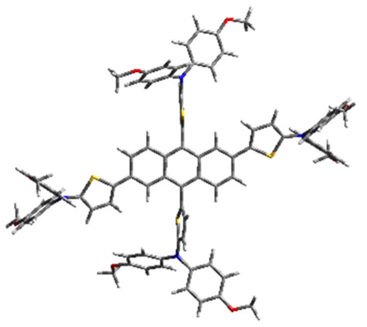</td><td>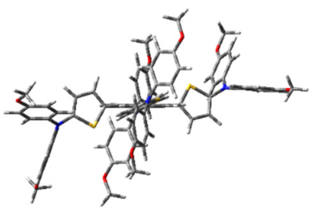</td></tr>
</table>

Figure S4. Calculated molecular geometries and dihedral angles between adjacent groups for **X1** and **X2**.

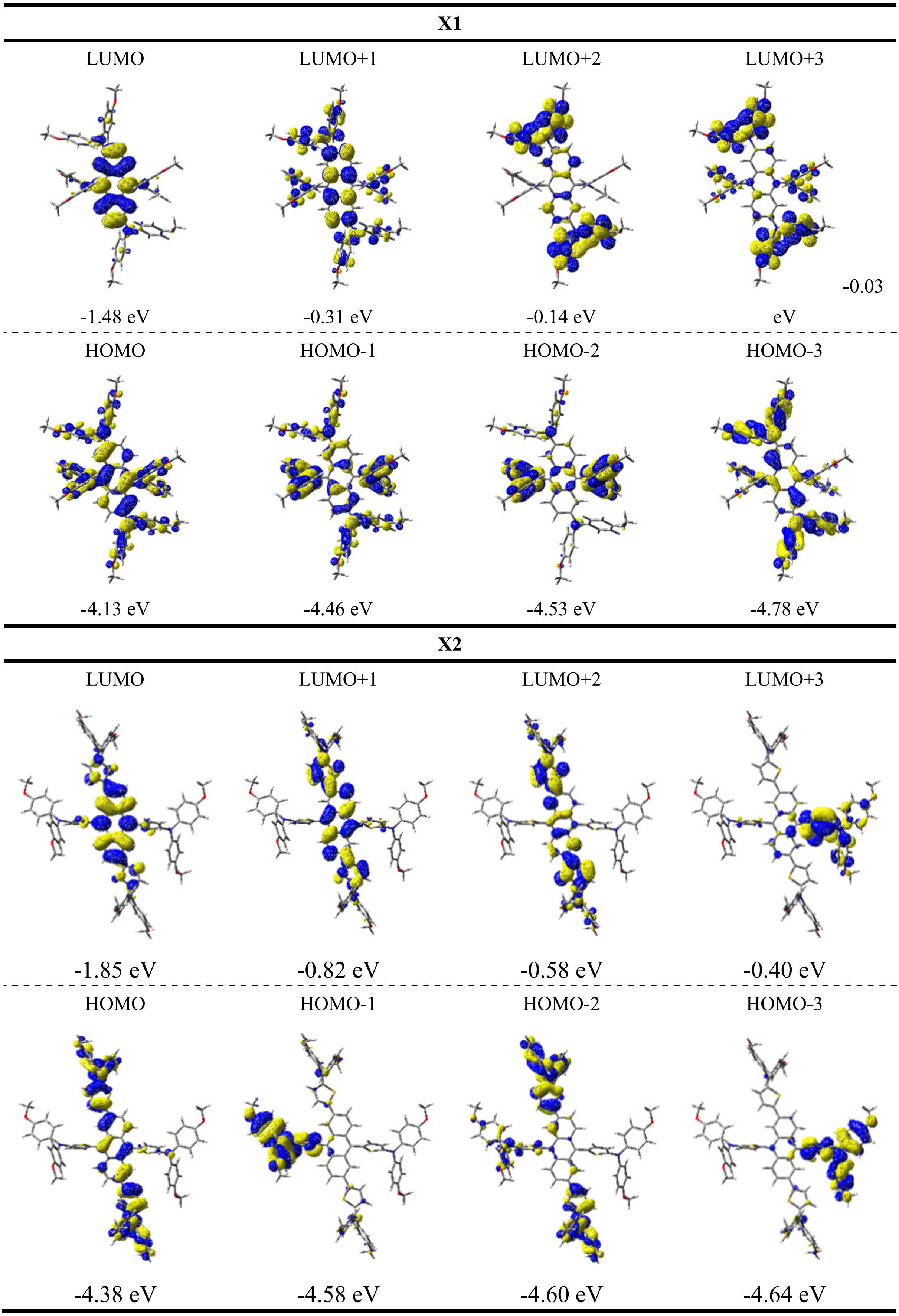

Figure S5. Frontier molecular orbitals for ground-state optimized **X1** and **X2**.

Table S1. Calculated TDDFT results at B3LYP/6-31G* level showing excitation energies (E_{cal}), oscillator strengths (f) and components for each vertical excitations.

	State	E_{cal}, eV	f	Components			State	E_{cal}, eV	f	Components	
**X1**	S1	2.19	0.137	H1→L	4%	**X2**	S1	2.25	0.576	H→L	49%
				H→L	45%						
	S2	2.43	0.007	H2→L	46%		S2	2.28	0.002	H1→L	49%
				H1→L	3%						
	S3	2.45	0.214	H2→L	3%		S3	2.38	0.016	H2→L	49%
				H1→L	42%						
				H→L	4%						
	S4	2.85	0.001	H3→L	49%		S4	2.52	0.016	H3→L	49%
	S5	3.25	0.219	H9→L	1%		S5	2.99	0.101	H4→L	41%
				H4→L	11%					H→L1	7%
				H→L1	35%						
	S6	3.47	0.513	H4→L	33%		S6	3.14	0.965	H5→L	1%
				H1→L1	4%					H4→L	7%
				H→L1	10%					H3→L1	2%
										H3→L2	2%
										H→L1	35%
										H→L2	1%
	S7	3.50	0.014	H→L2	46%		S7	3.33	0.032	H3→L1	3%
										H1→L1	34%
										H→L2	11%
	S8	3.59	0.040	H3→L2	2%		S8	3.36	0.026	H3→L1	7%
				H2→L2	1%					H1→L1	16%
				H1→L1	15%					H→L2	24%
				H→L3	29%						
	S9	3.63	0.009	H2→L1	19%		S9	3.44	0.005	H2→L1	47%
				H1→L1	1%						
				H1→L4	1%						
				H→L4	26%						
	S10	3.66	0.383	H4→L	3%		S10	3.49	0.127	H5→L	2%
				H1→L1	28%					H3→L1	33%
				H→L3	14%					H3→L2	1%
				H→L4	1%					H→L1	2%
										H→L2	9%

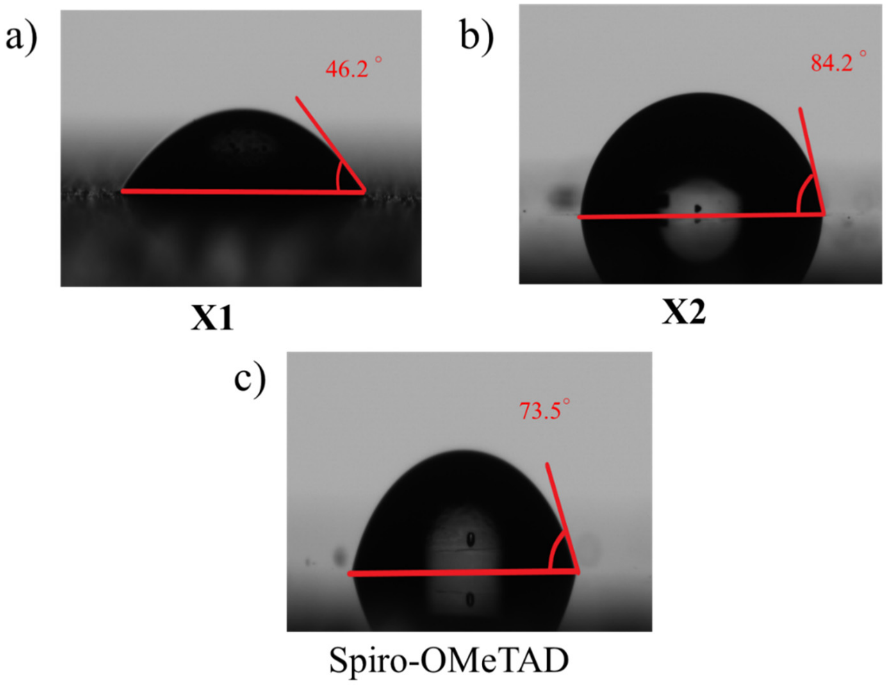

Figure S6. Contact angle between water and HTM film of (**a**) **X1**, (**b**) **X2**, and (**c**) spiro-OMeTAD on perovskite.

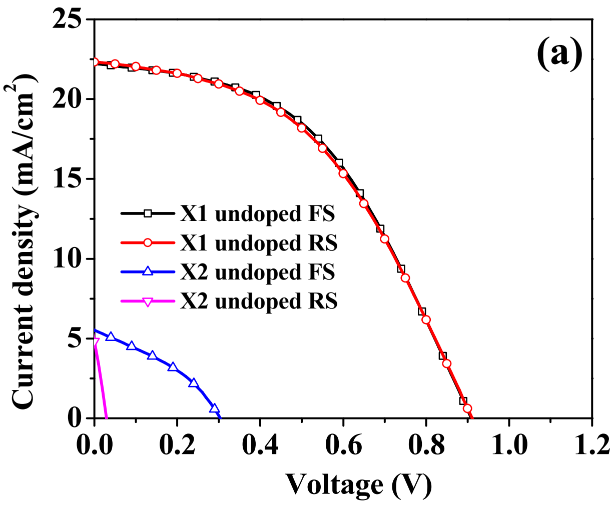

Figure S7. *J–V* curves of PSCs undoped **X1**, **X2** under forward and reverse scan measured under AM 1.5G illuminations in ambient atmosphere at a constant rate 100 mV s^{-1}.

Table S2. Device stability parameters of perovskite solar cells based on the **X1**, **X2**, Spiro-OMeTAD HTMs.

**HTM**		**direction**	V_{OC} (V)	J_{SC} (mA/cm^2)	*FF*	**PCE (%)**
X1(8h)	doped	FS	1.005	23.799	65.269	15.511
		RS	1.012	23.721	67.543	16.104
X1(12h)	doped	FS	0.979	23.394	64.828	14.758
		RS	0.984	23.493	64.087	14.715
X1(96h)	doped	FS	0.994	23.614	64.370	15.039
		RS	0.994	23.627	64.987	15.177
X1(204h)	doped	FS	0.919	20.549	66.208	12.500
		RS	0.943	20.855	67.961	13.855
X1(300h)	doped	FS	0.924	19.613	66.411	12.016
		RS	0.955	19.683	68.326	12.820
X2(8h)	doped	FS	0.933	16.432	52.763	8.08
		RS	0.936	16.568	66.165	10.25
X2(12h)	doped	FS	1.039	13.761	67.675	9.665
		RS	1.042	16.398	50.597	8.583
X2(96h)	doped	FS	0.903	14.245	55.472	7.112
		RS	0.848	14.940	59.031	7.470
X2(204h)	doped	FS	0.901	14.137	55.410	7.005
		RS	0.831	14.842	58.320	7.126
X2(300h)	doped	FS	0.854	12.839	41.506	4.551
		RS	0.925	13.641	47.925	6.048
spiro-OMeTAD(0h)	doped	FS	1.025	23.228	74.281	17.589
		RS	1.045	23.262	75.219	18.171
spiro-OMeTAD(8h)	doped	FS	1.079	23.022	73.056	18.031
		RS	1.088	22.934	75.012	18.587
spiro-OMeTAD(12h)	doped	FS	1.089	23.504	71.814	18.318
		RS	1.096	23.446	71.239	18.248
spiro-OMeTAD(96h)	doped	FS	1.002	23.318	70.325	16.340
		RS	1.022	23.265	71.967	17.025
spiro-OMeTAD(300h)	doped	FS	0.971	23.145	66.838	14.967
		RS	0.999	23.028	66.277	15.198

n-i-p device: ITO/SnO_2/ $CH_3NH_3PbI_3$/HTM/Au.

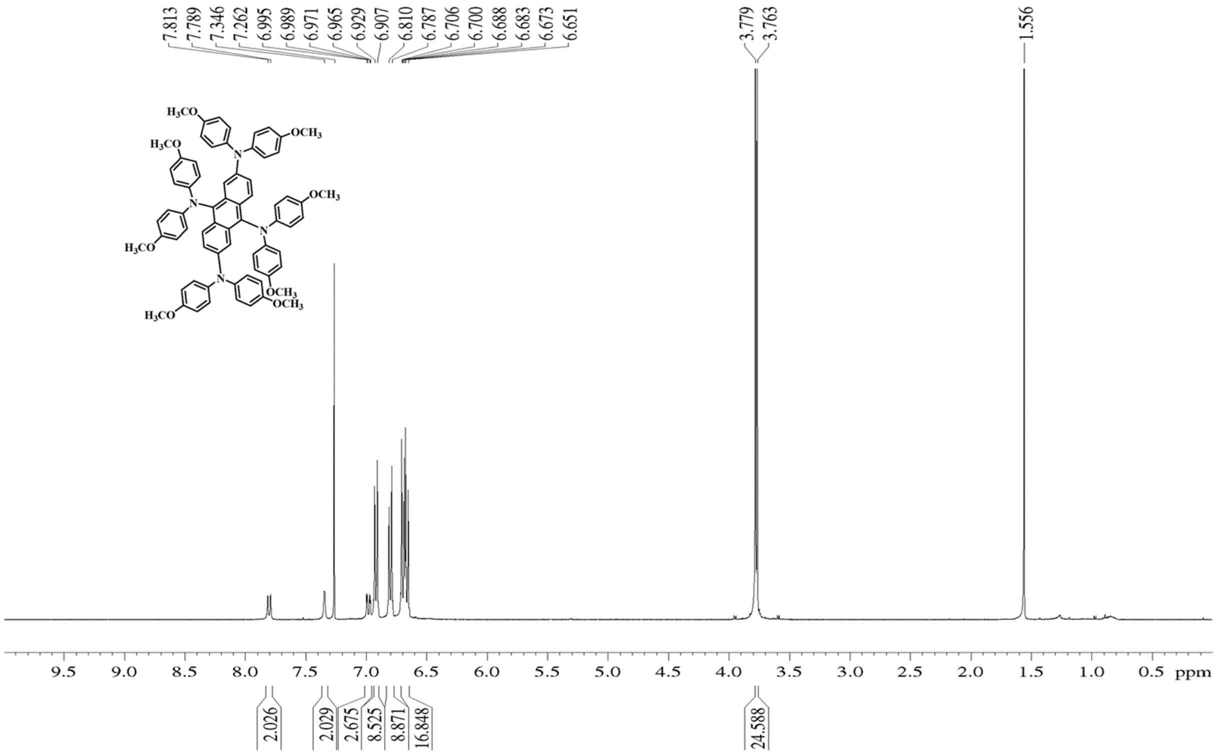

Figure S8. ^{1}H NMR spectrum of **X1** in $CDCl_3$.

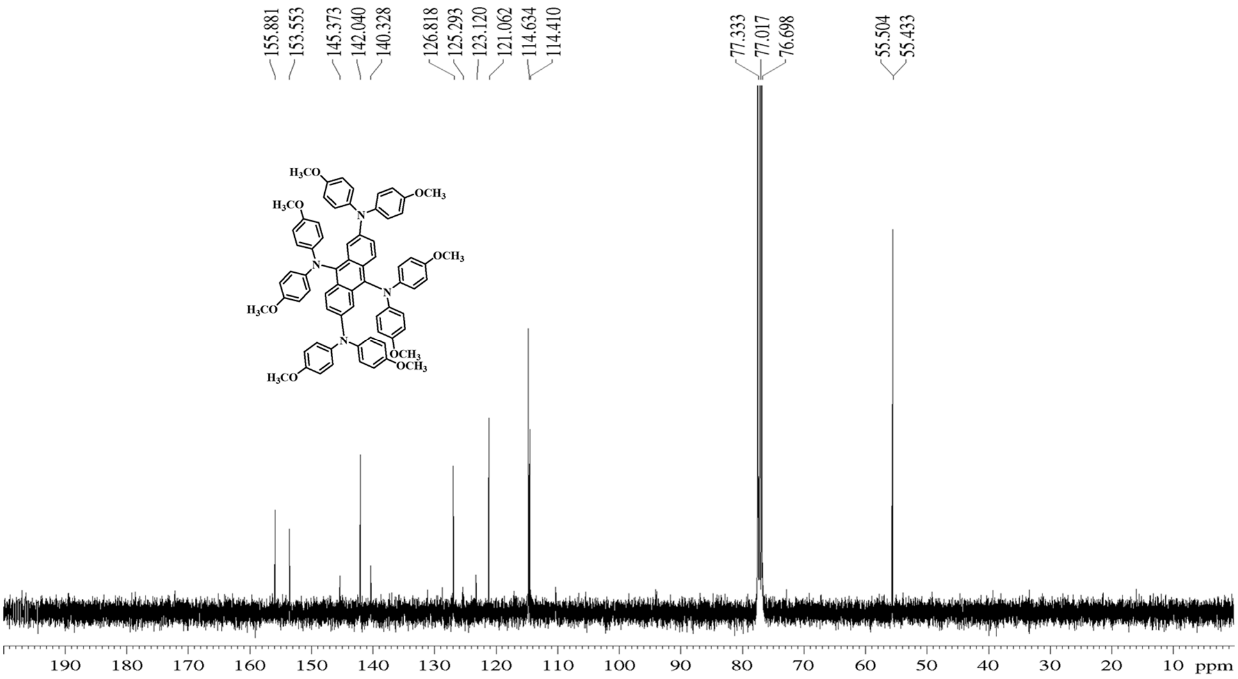

Figure S9. ^{13}C NMR spectrum of **X1** in $CDCl_3$.

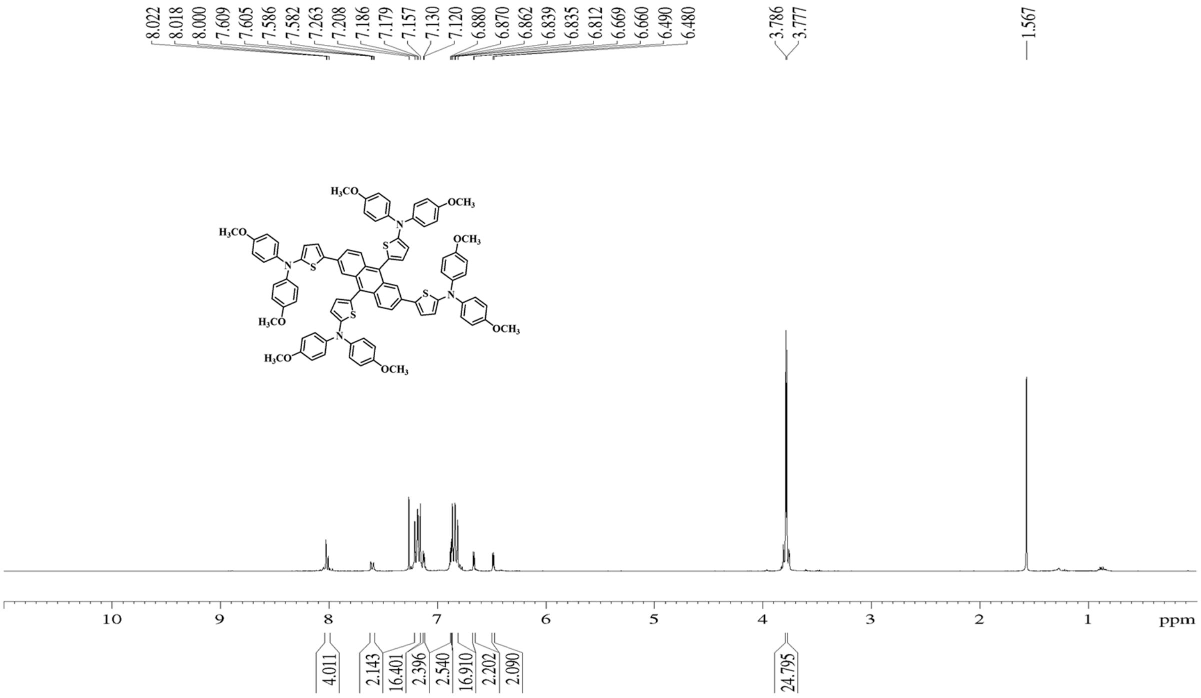

Figure S10. ^{1}H NMR spectrum of **X2** in $CDCl_3$.

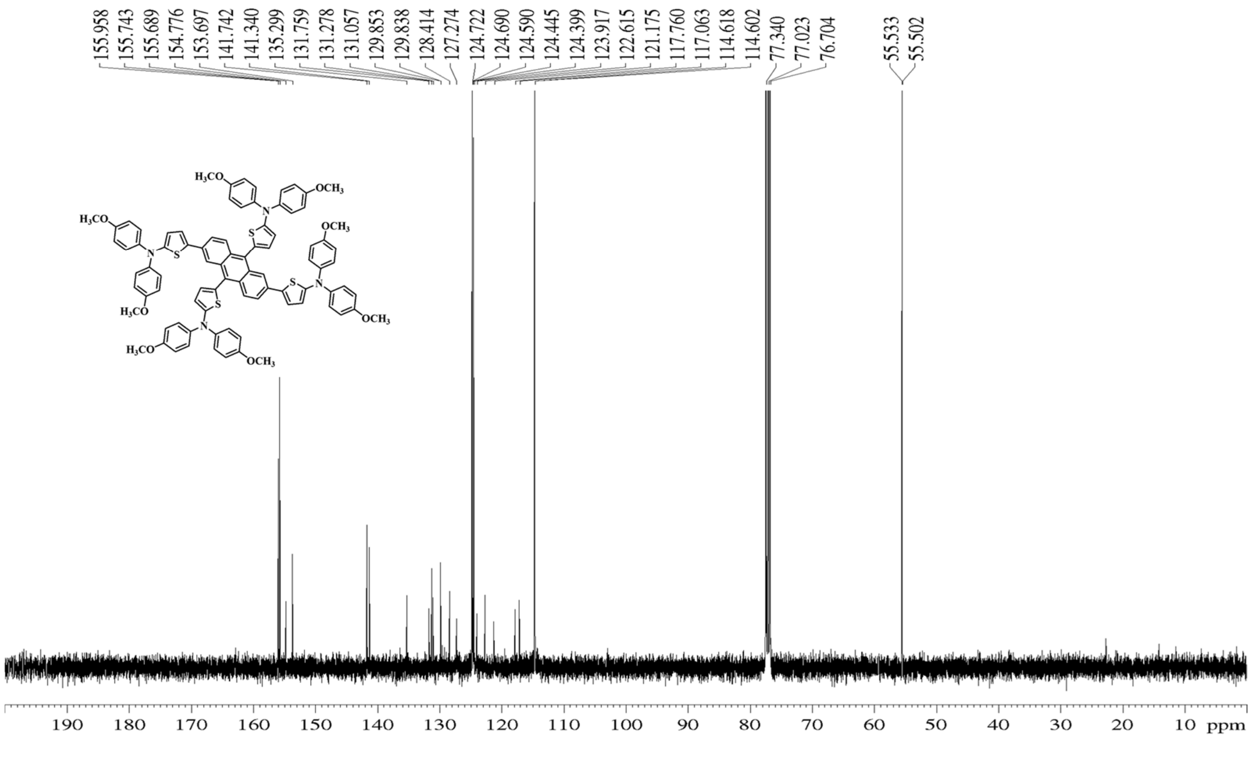

Figure S11. ^{13}C NMR spectrum of **X2** in $CDCl_3$.
